# Screening Antibodies Raised against the Spike Glycoprotein of SARS-CoV-2 to Support the Development of Rapid Antigen Assays

**DOI:** 10.1021/acsomega.1c01321

**Published:** 2021-07-27

**Authors:** Jason L. Cantera, David M. Cate, Allison Golden, Roger B. Peck, Lorraine L. Lillis, Gonzalo J. Domingo, Eileen Murphy, Bryan C. Barnhart, Caitlin A. Anderson, Luis F. Alonzo, Veronika Glukhova, Gleda Hermansky, Brianda Barrios-Lopez, Ethan Spencer, Samantha Kuhn, Zeba Islam, Benjamin D. Grant, Lucas Kraft, Karine Herve, Valentine de Puyraimond, Yuri Hwang, Puneet K. Dewan, Bernhard H. Weigl, Kevin P. Nichols, David S. Boyle

**Affiliations:** † PATH, 2201 Westlake Avenue, Suite 200, Seattle, Washington 98121, United States; ‡ Global Health Laboratories, 14360 SE Eastgate Way, Bellevue, Washington 98007, United States; § AbCellera Biologics Inc., 2215 Yukon Street, Vancouver, BC V5Y 0A1, Canada; ∥ Intellectual Ventures Lab, 14360 SE Eastgate Way, Bellevue, Washington 98007, United States

## Abstract

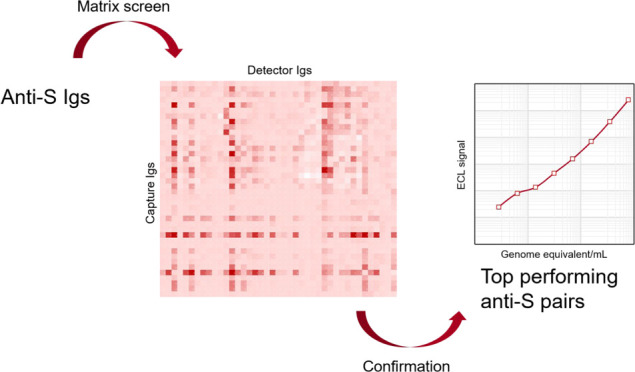

Severe acute respiratory coronavirus-2 (SARS-CoV-2) is a novel viral pathogen and therefore a challenge to accurately diagnose infection. Asymptomatic cases are common and so it is difficult to accurately identify infected cases to support surveillance and case detection. Diagnostic test developers are working to meet the global demand for accurate and rapid diagnostic tests to support disease management. However, the focus of many of these has been on molecular diagnostic tests, and more recently serologic tests, for use in primarily high-income countries. Low- and middle-income countries typically have very limited access to molecular diagnostic testing due to fewer resources. Serologic testing is an inappropriate surrogate as the early stages of infection are not detected and misdiagnosis will promote continued transmission. Detection of infection via direct antigen testing may allow for earlier diagnosis provided such a method is sensitive. Leading SARS-CoV-2 biomarkers include spike protein, nucleocapsid protein, envelope protein, and membrane protein. This research focuses on antibodies to SARS-CoV-2 spike protein due to the number of monoclonal antibodies that have been developed for therapeutic research but also have potential diagnostic value. In this study, we assessed the performance of antibodies to the spike glycoprotein, acquired from both commercial and private groups in multiplexed liquid immunoassays, with concurrent testing via a half-strip lateral flow assays (LFA) to indicate antibodies with potential in LFA development. These processes allow for the selection of pairs of high-affinity antispike antibodies that are suitable for liquid immunoassays and LFA, some of which with sensitivity into the low picogram range with the liquid immunoassay formats with no cross-reactivity to other coronavirus S antigens. Discrepancies in optimal ranking were observed with the top pairs used in the liquid and LFA formats. These findings can support the development of SARS-CoV-2 LFAs and diagnostic tools.

## 1 Introduction

The appearance of a novel coronavirus disease 2019 (COVID-19) was first reported in the city of Wuhan, Hubei Province, China in 2019.^1^ As of March 11, 2021, the COVID-19 pandemic has continued to progress with over 178 million reported cases including over 3.8 million associated deaths globally.^2^ The pathogen responsible is the severe acute respiratory syndrome coronavirus-2 (SARS-CoV-2), a novel betacoronavirus. These coronaviruses are enveloped positive-stranded RNA viruses that are 70–90 nm in size and characterized by a crownlike morphology associated with the display of spike (S) glycoproteins on the host membrane-derived and lipid bilayer viral envelope.^3,4^ The structure of the S glycoprotein of SARS-CoV-2 has been resolved and is known to be essential for the viral infection of the host cell via its binding to the cellular receptor angiotensin-converting enzyme 2 to promote fusion and entry into the cell.^5^ The S glycoprotein is poorly conserved across coronaviruses, with 85.3% of the antibody epitopes found in SARS-CoV-2 S protein considered unique.^6,7^ Conversely, higher conservation noted across SARS-CoV-2 isolates from Europe, Asia, and the United States, resulting in an antigen that offers greater specificity over more conserved targets like nucleocapsid (N) antigen.^8,9^

The rapid spread of COVID-19 has resulted in an urgent need for effective diagnostic tests to support disease management, monitoring, surveillance, and pandemic control against SARS-CoV-2.^10^ In high-income countries, molecular testing, typically using real-time reverse transcription polymerase chain reaction (RT-PCR), has been the primary test method implemented to diagnose SARS-CoV-2 in both symptomatic and asymptomatic cases, but the accurate detection of early infection remains challenging due to the possibility of false negative results.^11,12^ As of December 30, 2020, 315 commercial or clinical laboratory-derived molecular tests have been granted emergency use authorization (EUA) in the United States by the Food and Drug Administration (FDA).^13^ Many of the tests are predominantly unsuitable for use at the point-of-care as many are in open assay format with which significant engagements are needed from skilled operators working in dedicated laboratory spaces to prepare samples for testing, prepare test reagents, operate complex equipment, and finally to process the data and interpret the test results. Automated high-throughput molecular platforms are available and are capable of processing large numbers of samples with significantly reduced operator input.^14−17^ However, acquiring and operating such equipment comes with high capital costs and a need for appropriate infrastructure, not only for housing the equipment and reagents but also requiring effective specimen collection and transport and the reporting of test data to patients, clinicians, and health care programs after processing. In the current pandemic, global demand has affected all countries and so sufficient access to reagents, consumables, and other materials such as personnel protective equipment, swabs, and transport media is necessary to ensure consistent testing.^18^

Lack of access to key reagents and consumables has highlighted that there is a market for SARS-CoV-2 diagnostic immunoassay-based lateral flow assays (LFAs) in high-income countries. Low- and middle-income countries (LMICs) already faced serious constraints in diagnostic capacity and accessibility before the COVID-19 pandemic struck. SARS-CoV-2 will have an amplified effect in these countries that have limited access to care with an already greater burden of infectious diseases.^19^ LMICs lack time and finances for the swift uptake of new diagnostic technologies. Furthermore, a lack of resources and skilled laboratorians limits the number of test facilities and the ability to scale testing, while access to critical reagents is limited as high-income countries dominate procurement, culminating in the inability to perform molecular tests at the scale required.^20^ Without access to expanded molecular test capacity and capability, other diagnostic tools must be developed to support COVID-19 infection control. Therefore, LFAs serve as the best alternative in regions lacking sufficient access to widespread molecular testing for SARS-CoV-2.

For detection and control of COVID-19 in LMICs, an antigen LFA format makes a more viable option to the serologic LFAs that currently dominate the market due to their ability to detect SARS-CoV-2 directly and earlier in the infection process. Serology-based assays are insensitive in early infection requiring individuals to be diseased for at least a week before the antibody response can first be detected (IgA, IgM, and/or IgG),^21^ which is enough time for an infected individual to unknowingly spread the disease.^22^

The performance of antigen detection LFAs is variable depending on the performance of the antibodies used in the test, and while visually read LFA reach the level of sensitivity that molecular assays offer, the use of readers can further increase test sensitivity. The recent FDA EUA to Lumira Diagnostics (Stirling, U.K.) for their SARS-CoV-2 Ag assay has claims of a sensitivity of 97.6% as compared to RT-PCR testing. Therefore, rapid antigen assays using high-performance antibodies can offer sufficient clinical sensitivity to detect infectious patients in decentralized settings, where molecular testing is not readily available. Furthermore, LFAs can be manufactured at extremely high volumes and very low costs and can offer increased testing capacity in LMICs. Other markets where LFAs can play a key role are in disseminated testing models such as community- and home-based testing, and self-testing.^23−25^

The WHO’s recently released target product profile for a point-of-care test for suspected COVID-19 cases (e.g., a rapid antigen assay) has listed the acceptable characteristics for sensitivity and specificity at ≥70 and ≥97%, respectively.^26^ A current challenge to antigen test development is understanding the performance of SARS-CoV-2 antibodies that are on or entering the market, with the screening of large numbers of unqualified antibodies a resource sink for developers aiming to develop direct antigen tests. Abundant targets include the four major structural proteins: the spike (S), membrane (M), envelope (E), and nucleocapsid (N) proteins. The S glycoprotein represented an attractive candidate due to the unique structural changes relative to SARS-CoV-1 and other seasonal coronaviruses, offering the potential of high specificity for SARS-CoV-2.^6^

In recent studies, we assessed the performance of anti-N protein antibodies via half-strip LFAs.^27^ While a lower prevalence target than the N, the structural role of S may present better epitopes to antibodies and so could be an attractive target for a rapid LFA.^27,28^ In this study, we accessed multiple antibodies targeting the S glycoprotein by leveraging the antibody therapeutics industry and commercially available sources. We assessed their performance using recombinant S antigens and inactivated cultured SARS-CoV-2 virus and S glycoproteins from other human coronavirus species, using a highly sensitive liquid immunoassay in addition to a high-throughput half-strip LFA screen.^27,29^ These screens enabled us to down select and identify the optimal pairs that offer the greatest sensitivity and specificity for further development and incorporation into liquid and LFA immunoassay formats for direct antigen detection of SARS-CoV-2 virus via the S glycoprotein.

## 2 Results and Discussion

### 2.1 Liquid Immunoassay Screening

All data generated from this screening is publicly accessible.^30^ A total of 48 monoclonal antibodies (AbCellera, 41; Sino Biological, 3; and Leinco, 4) were assessed for their performance as capture and detection antibodies for the SARS-CoV-2 S glycoprotein across three rounds of testing using the Meso Scale Discovery (MSD) U-PLEX assay platform. Each well in a 96-well U-PLEX plate can host 10 different capture antibodies per well in a geometric planar array (960 capture events per plate). The assay enabled rapid screening of multiple antibody combinations to identify the most promising candidate pairs that would enable sensitive and specific capture and detection of SARS-CoV-2.

**Figure 1:**
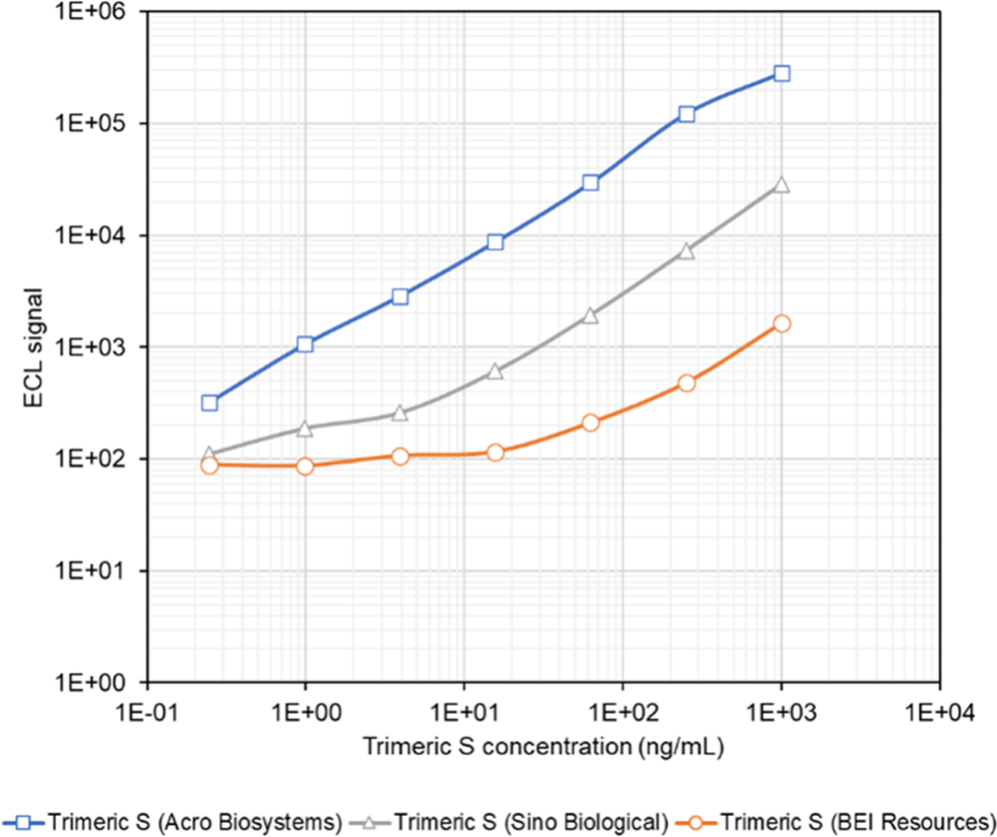
Curves demonstrating the assay performance of three commercially available trimeric S glycoproteins across a range of dilutions when screened using the AbC525–AbC397 pair. LOD_Acro Biosystems_ = 286 pg/mL, LOD_Sino Biological_ = 768 pg/mL, and LOD_BEI_ = 19665 pg/mL.

In the preliminary evaluations, a recombinant S glycoprotein antigen expressed from insect cells (BEI) was used to screen AbCellera antibodies. However, this antigen resulted in the generation of very low electrochemiluminescence (ECL) signals at the concentration used. We postulated that post-translational modifications during antigen production differ between insect cells and mammalian cells, and the antigen initially used may have had or may have lacked modifications that made it unsuitable for our study.^31^ To identify an antigen most suitable for this work, we evaluated three recombinant S glycoproteins across a range of concentrations (1000–0.24 pg/mL) using AbC525 and AbC397 as capture and detector, respectively, as this pair had generated the strongest ECL in the preliminary screen. The signal intensities and limit of detection (LOD) varied with respect to each of the three antigens used. The mammalian cell-derived recombinant S glycoprotein from Acro Biosystems produced the strongest and more consistent signal as compared to baculovirus expressed antigens and the lowest LOD (Figure 1). Thus, this antigen was selected for use as the standard in all liquid immunoassay screens.

**Figure 2:**
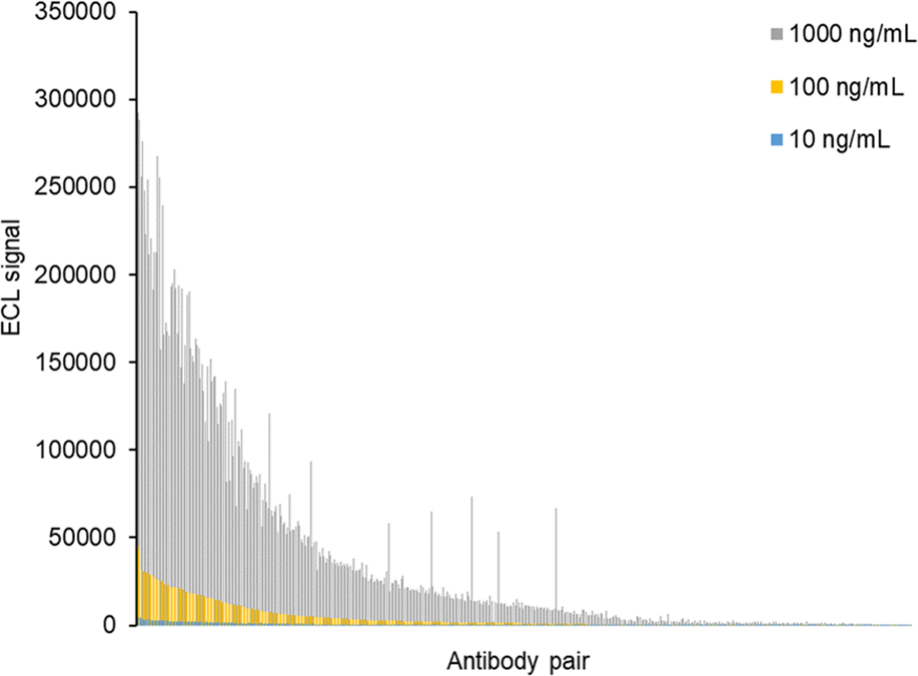
460 AbCellera antibody pairs, sorted based on ECL signals at 10, 100, and 1000 ng/mL of trimeric S glycoprotein antigen. ECL, electrochemiluminescence.

In round 1, 41 antibodies from AbCellera were assessed in both capture and detector format using a low S glycoprotein antigen concentration of 10 ng/mL to allow for more stringent screening. One antibody, AbC298, failed to biotinylate even after three attempts and was not evaluated as a capture antibody. In total, there were 1640 antibody pairs assessed in this round; each pair exhibited varying affinity to S glycoprotein. Table 1 summarizes the round 1 screening results in a matrixed array for each antibody combination. In the absence of a positive control assay, the ECL values from each array spot in each well were normalized based on the percentile of signal-minus-noise (S-N) in each plate versus the spot with the maximum S-N produced in each plate. A total of 117 (7.0%) antibody pairs produced at least 25% of the maximum signal (marked in gray and dark gray boxes). These pairs, consisting of 20 capture and 23 detection antibodies, were further screened in a total of 460 combinations with S antigen in a range of 10, 100, and 1000 ng/mL to confirm the initial results (Figure 2). The 10 antibody pairs that generated the highest ECL were selected for evaluation in round 2 and included two capture antibodies (AbC447 and AbC525) and five detector antibodies (AbC513, AbC518, AbC459, AbC447, and AbC511), with AbC447/AbC513 capture/detector pair having the highest ECL. No self-pairing antibodies were identified presumably due to the presence of only a single epitope on the recombinant antigen that limits binding to only one form of the respective labeled antibodies.

**Table 1:**
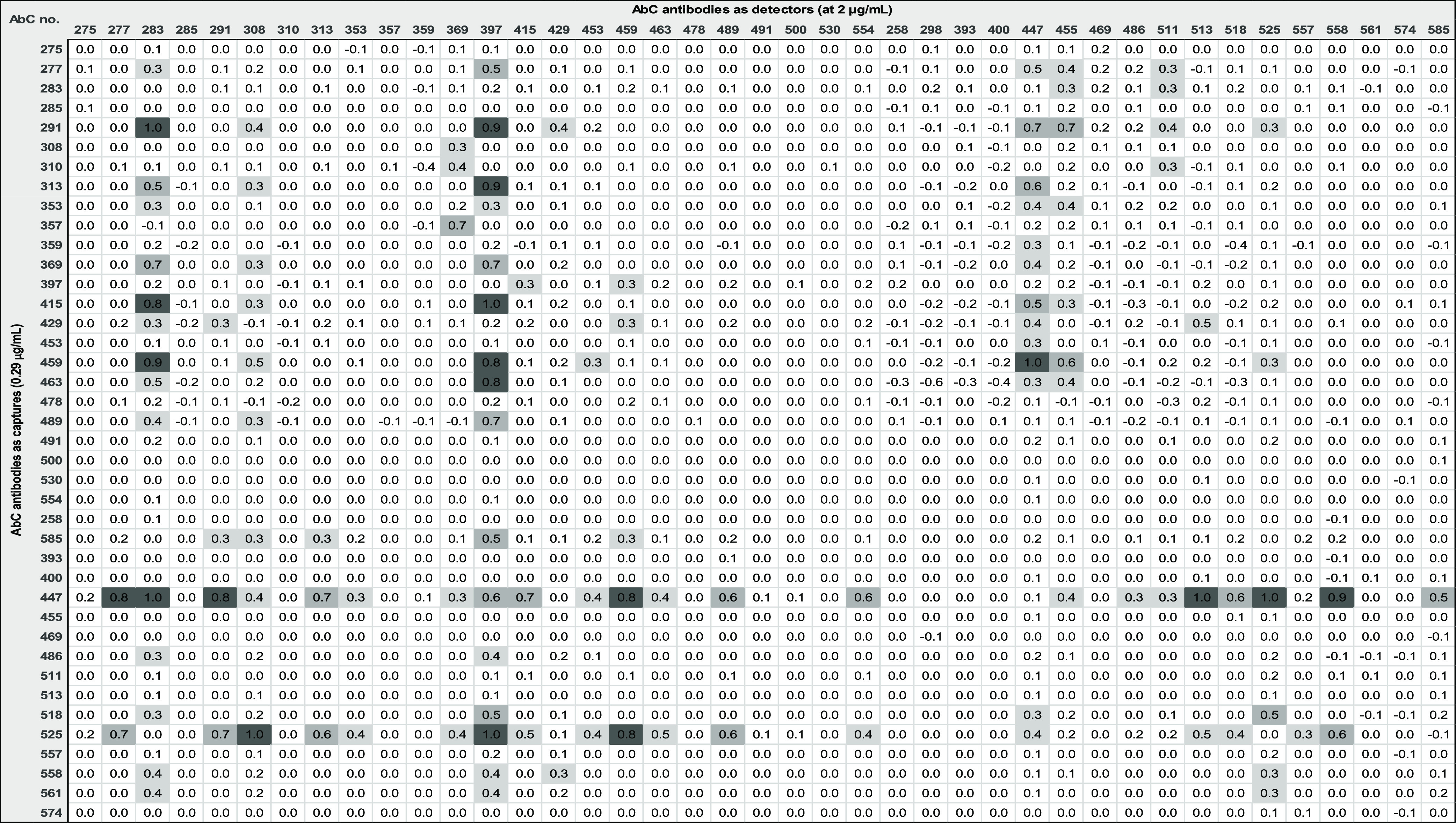
Performance of the AbCellera Antibodies in Capture (*n* = 40) and Detector (*n* = 41) Formats in Sandwich Assays^a^

a The color gradient represents antibody pair performance, measured as signal-noise. A darker shade indicates a pair performed better. Numbers inside the grid are normalized 0–1.0 according to the pairs with lowest and highest S-N. Legends: dark gray box <0.75–1.00, medium gray box <0.50–0.74, light gray box <0.25–0.49, and white box 0.00–0.24.

In round 2, the 10 optimal AbCellera antibody pairs were assessed further in a matrix format alongside three antibodies from Sino Biological (MM43, MM57, and D003). Screening with a seven-point standard curve indicated that the Sino Biological antibodies resulted in higher ECL signals and lower LODs than the best AbCellera pair (AbC447/AbC513) (Table 2). Notably, the Sino 447/MM43 and 447/D003 pairs exhibited similarly low LODs at 43 and 45 pg/mL, respectively, in addition to the highest ECL signals when challenged with ≥ 625 ng/mL of trimeric S antigen. Antibody pairs AbC447/MM43 and AbC447/D003 were then further challenged with a range of concentrations of SARS-CoV-2 virions, both detecting down to 80 TCID_50_/mL or approximately 4.86 × 10^5^ genome equivalents/mL; the AbC447/MM43 pair was determined to have the best performance characteristics at this stage.

**Table 2:** Top Antibody Pairs from Rounds 2 and 3 when Challenged with S Glycoprotein Ranked Based on Their LOD as a Selection Criterion^a^

capture Ig	detector Ig	ECL signal (625 ng/mL antigen)	LOD (pg/mL)
Round 2 Top 5 Performers			
AbC447	MM43	801 003	43
AbC447	D003	871 186	45
AbC447	AbC513	215 672	71
D003	MM43	168 029	94
MM43	AbC447	199 763	174
Round 3 Top 5 Performers			
L2381	MM43	1 097 669	3
L2355	L2215	2 200 950	4
L2838	L2215	2 580 454	6
L2381	L2215	1 760 625	7
L2355	MM43	1 568 224	8

a The electrochemiluminescent (ECL) signal generated with 625 ng/mL S antigen is also included.

**Figure 3:**
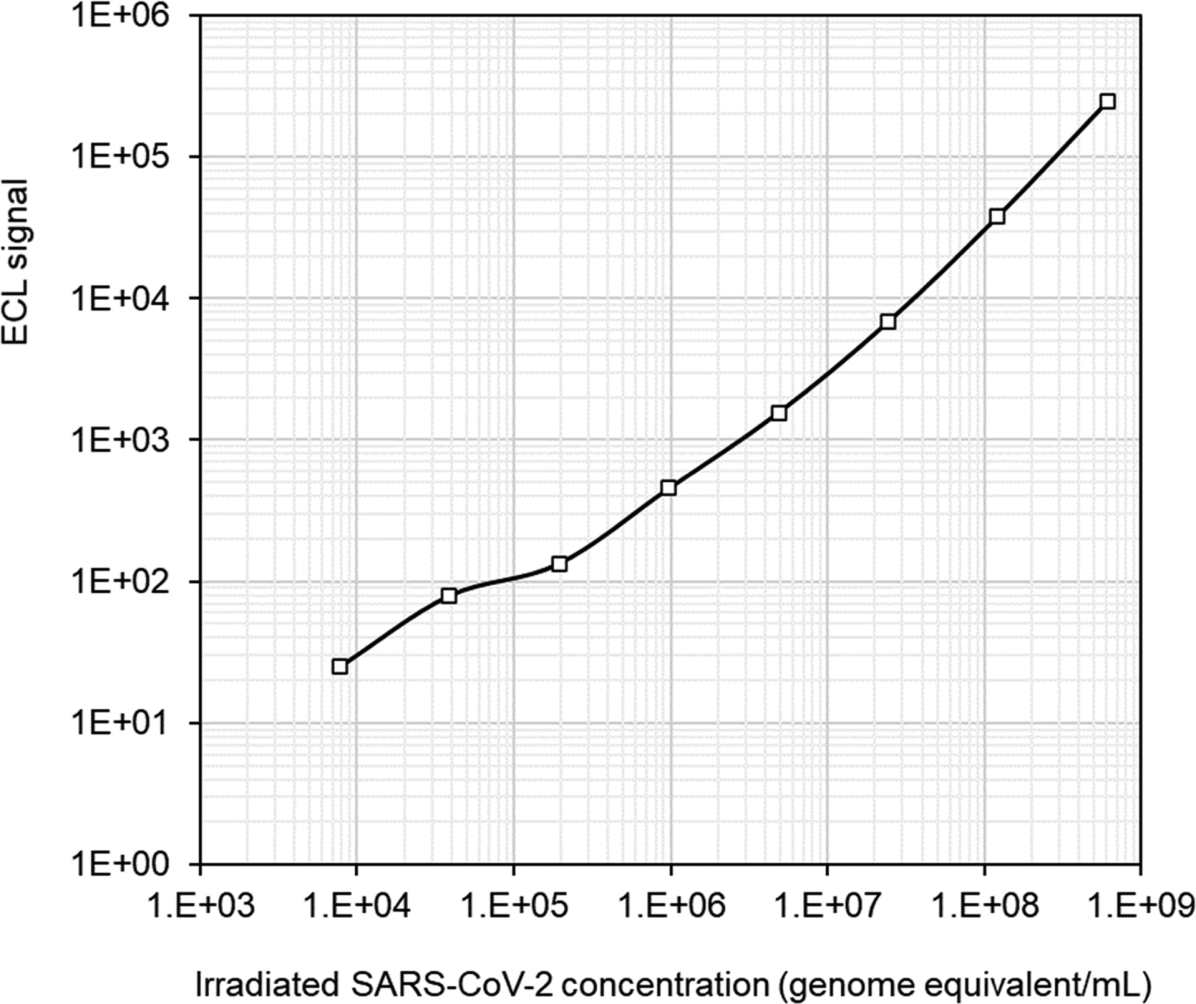
Detection of serial dilutions of inactivated SARS-CoV-2 using the L2355 (capture)/L2215 (detector) antibody pair.

In round 3, four antibodies from Leinco (L2215, L2355, L2381, and L2838) were screened with the six top antibody candidates identified from round 2 (D003, MM42, AbC447, and AbC513) and round 1 (AbC353 and AbC525). When used either as a capture or detector, the Leinco antibodies typically generated higher ECL signal and lower LODs than previously observed (Table 2), many with the LOD generally 5–10 times lower than for the best-performing antibody in round 2. The L2381/MM43 and L2355/L2215 combinations had near-identical LODs at 3 and 4 pg/mL, respectively, with L2355/L2215 selected for further study due to greater affinity to the target as indicated by significantly higher ECL signal when challenged with S antigen at 625 ng/mL (Table 2). The antibody pair L2355/L2215 was challenged with a titered SARS-CoV-2 (BEI), resulting in the generation of a dose-dependent curve (Figure 3) with an estimated LOD of 2 TCID_50_/mL virions or 7.4 × 10^3^ genome equivalents/mL.

To demonstrate assay performance of the L2355/L2215 antibody pair with clinical samples, a panel of 53 deidentified clinical samples, comprising 20 COVID-19 negatives and 33 COVID-19 positives, were used to challenge the assay. Of these, 44 of the 53 samples were correctly identified as either positive or negative (Table 3). The viral load of the specimen was important as nine positive samples, each with a cycle threshold of >29.5, were incorrectly scored as negative. This was likely in part due to dilution of the sample as each nasopharyngeal swab was collected in 3 mL of viral transport medium. Overall, the assay had a sensitivity and specificity of 73 and 100%, respectively (Table 3), when compared to the RT-PCR results.

**Table 3:** Performance Characteristics of the L2355 (Capture)/L2215 (Detector) Pairing Relative to qRT-PCR When Challenged with 53 Clinical Specimens

	MSD +ve	MSD −ve	total	sensitivity (%)	specificity (%)
RT-PCR +ve	24	9	33	73	100
RT-PCR −ve	0	20	20		
total	24	29	53		

The cross-reactivities of the antibody pairs from rounds 2 and 3 (Table 2) were also evaluated by challenging them with α- and β-coronavirus isolates, including inactivated Middle East respiratory syndrome virus (MERS) and SARS-CoV virions and human coronaviruses OC43 and 229E cell culture lysates at concentrations equivalent to 10^4^ TCID_50_/mL or 10^4^ PFU/mL. None of the 10 antibody pairs showed any cross-reactivity with other human CoV, indicating a high specificity toward SARS-CoV-2.

### 2.2 Candidate Screening via Lateral Flow Assays

**Figure 4:**
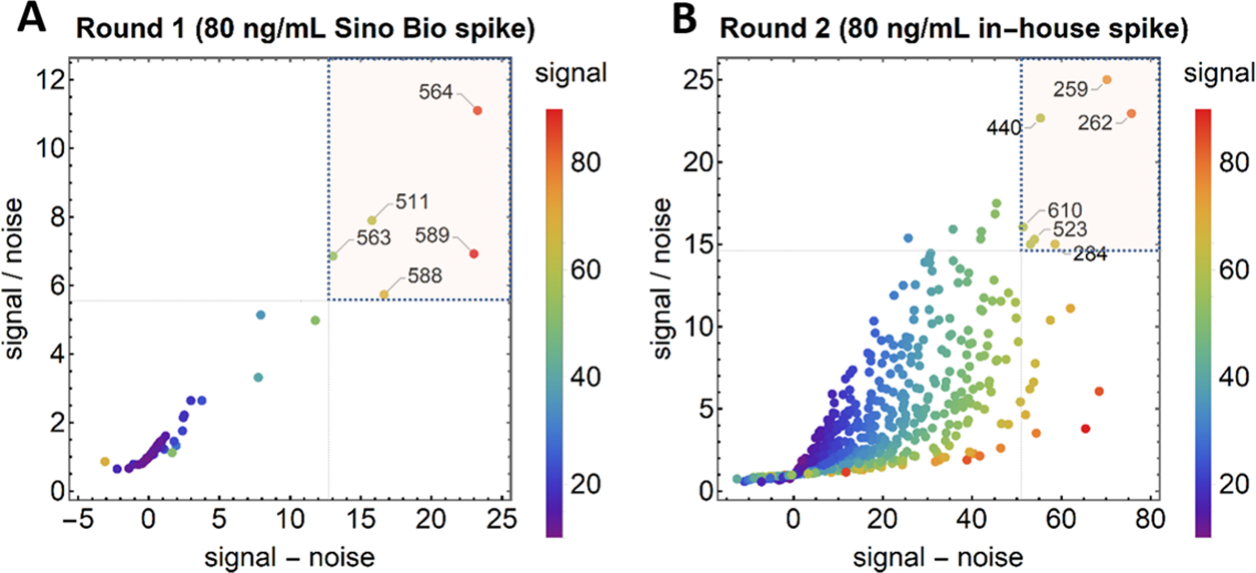
Performance of 621 individual antibody pairs in two rounds of screening on the LFA format as a function of signal/noise and signal-noise. Line intensities are shown as scatter plots for round 1 (A) and round 2 (B). Antibody pairs performing in top 5 [for average rank by S/N and S-N] are overlaid with a semitransparent box and numbered by their index (full list in Table S2). “Sino Bio spike” was sourced from Sino Biological and “in-house spike” recombinant antigen was produced and purified at Global Health Labs.

The candidate antibodies were also evaluated in the LFA format in two rounds of screens, to assess if the performance of the candidate antibodies varied between the liquid and LFA test formats. A total of eight antibodies (AbC131 from AbCellera, D003 from Sino Biological, and six other antibodies from Sino Biological and Creative Diagnostics) were evaluated in round 1 on LFAs in an 8 × 8 matrix (64 unique pairs, see Table S1). For each pair, one antibody was striped on nitrocellulose as a test line (the “capture” antibody) and the other was coupled to latex nanoparticles using 1-ethyl-3-(3-dimethylaminopropyl)carbodiimide/*N*-hydroxy succinimide (EDC/NHS) chemistry (the “detector” antibody). The results of the first round are given in Figure 4A. The positive control used in round 1 was 80 ng/mL S glycoprotein from Sino Biological, selected due to the presence of both the S1 and S2 domains of the native spike trimer. The negative control was 2.5% bovine serum albumin (BSA) in PBS-T.

After the first round, the best five pairs were D003/D002, D004/D002, D001/D004, D004/D001, and D003/D001 (indices 564, 589, 511, 568, and 563, Table 3). Each of the top pairs from round 1 consisted exclusively of antibodies from Sino Biological, which was unsurprising considering recombinant antigen choice and the fact that most antibodies screened were from Sino Biological. As with the liquid immunoassay screen, self-pairs did not perform well, as expected, a consequence of the monomeric recombinant antigen likely containing a single copy of the target sequence. However, we would expect self-pairs to do better against the native antigen in clinical samples because it is trimeric. After round 1, 57 anti-S pairs were eliminated and the top 7 pairs were carried to round 2, along with 22 new antibodies. These new antibodies included the 12 top performing AbCellera antibodies from round 1 liquid immunoassay screen, MM43 from Sino Biological, and nine antibodies from Leinco Technologies, including the four antibodies already screened with liquid immunoassay (Figure S1; found in Supporting Information documents).

The grid for round 2 was larger at 26 × 26 (616 pairs); however, limited access to material meant 60 pairs were ultimately excluded (Figure S1). Results from round 2 are shown in a scatterplot in Figure 4B. The positive control used here was a trimeric spike glycoprotein produced in-house, considered superior to the recombinant form due to its ability to better mimic the protein folding seen in native structures. The negative control used was 2.5% BSA in PBS-T. Based on signal-to-noise ratio (S/N) and S-N metrics, the five best-performing antibody indices from round 2 were 259, 262, 440, 284, and 523 (Table 4).

**Table 4:** Antibody Pairs in the Top 5 for Both the Signal-to-Noise Ratio [S/N] and Signal Minus Noise [S-N] Are Ranked According to the Round in Which They Were Tested^a^

			average rank	
index	capture	detector	rd. 1	rd. 2
Round 1 Top 5 Performers				
564	D003	D002	1	302
589	D004	D002	2.5	
511	D001	D004	3	
588	D004	D001	4	
563	D003	D001	4.5	156.5
Round 2 Top 5 Performers				
259	AbC459	L2355		1.5
262	AbC459	D001		1.5
440	L2355	AbC459		5.5
284	AbC525	L2355		9
523	D002	AbC459		11

a Table S2 (Supporting Information) contains a complete list of all pairs screened.

## 3 Summary and Conclusions

In this paper, we present the screening of a panel of antibodies targeting the S glycoprotein of SARS-CoV-2 to identify candidate capture and detector pairs that may be suitable for the development of LFA antigen detection assays. We gained access to a large private collection but with limited access to sufficient materials resulting in some antibodies being screened in one assay format and not the other. Commercially available antibodies were typically screened in both formats. A key to this work is the availability of a good native antigen proxy, and as antigen sources can vary considerably, it is important to assess them prior to commencing work. Using the highly sensitive MSD immunoassay platform, we were able to achieve an analytical sensitivity in the range of 7.4 × 10^3^ genomic copies/mL and a specificity of 100% when using a limited specimen panel. The target product profile for a test for diagnosis or confirmation of acute or subacute SARS-CoV-2 infection, suitable for low- or high-volume needs, notes a sensitivity of under 1000 copies, which this test does not currently meet. However, the intent of this project was to screen antibodies that have the optimal potential for implementation in LFAs, and not to develop a diagnostic assay. If necessary, the platform can use a further enhancement signal format not used here, the S-PLEX, which MSD claims can further improve sensitivity by 10–100X or into the lower femtogram range. While the S glycoprotein is less abundant than the N protein, there may be a utility for combining S as a target to create highly sensitive multiplex immunoassays, with its additional distinct epitopes enabling improved accuracy, especially at lower limits of detection.^32,33^

The liquid assays identified pairs that gave an analytical sensitivity to the S antigen into the low picogram range, a 10-fold improvement over previous N immunoassays reported for SARS-CoV, but the ECL detection feature of the MSD device does also offer greater sensitivity over traditional colorimetric detection employed by most enzyme immunoassay methods.^32,34^ Interestingly, the assay format had a distinct effect on the optimal candidate pairs identified. The L2355 and L2215 clones were the best antibodies in either platform and as both capture and detector. In contrast, no AbCellera antibodies showed good performance in the liquid assay, though in the LFA format AbC459 was present as a capture or a detector in 4/5 top pairs. The use of a different source of recombinant antigen may have played a role in this, as we did observe some differences in binding using mammalian recombinant sources of antigen. This finding serves as an insight into LFA developers, wherein screening of all antibodies should be performed on nitrocellulose rather than using traditional liquid immunoassays. The best antibody candidates screened in the liquid format appeared to be highly specific to SARS-CoV-2 as they were not reactive with SARS, MERS, or OC43 HCoVs that are in the same genus as SARS-CoV-2.^35^ While we did not have access to HKU1, another β-CoV species associated with respiratory illness, we do expect it is unlikely to be reactive as the other more closely related β-CoVs screened were nonreactive.

On the LFA platform, the best pairs, as measured by S/N and S-N, were from a combination of vendors (e.g., AbCellera, Leinco, and Sino Biological), likely because these high-affinity antibodies were raised via unique processes and therefore recognize different epitopes on the antigen.

Interestingly, liquid and LFA formats did identify very different optimal pairs for the detection of the S antigen. Restricted resources meant that entire antibody sets could not be fully evaluated on both platforms, but it was evident that some pairs were better suited to one format over the other. In the liquid format, none of the AbCellera antibodies were in the top candidates as either capture or detector by round 3, but with the LFA, AbC459 and AbC525 were represented in several optimal pairings (Table 2).

With the Sino Biological antibodies, a similar trend was noted wherein no candidates shone with the liquid immunoassay format, while the LFA had two, D001 and D002 (Tables 2 and 3). Antibodies from Leinco were highly represented in the optimal liquid assay design with each of the top 5 pairs having at least one Leinco antibody in the pairing. By contrast, with the top 5 candidates in the LFA format, three pairs used a single Leinco antibody, L2355, either as capture or detector, though in combination with different antibodies to the liquid format. This is primarily due to differences in the kinetics of antibody–antigen binding. In the liquid immunoassay, the contact time between antibodies and analytes is longer (up to 1 h), and therefore, the binding kinetics can be less important. Antibodies with faster on-rate (basically how quickly the antibody binds to the antigen to form a complex), however, have a greater impact for LFAs as the time the analyte spends in contact with the capture and detection antibodies is typically very brief (seconds to minutes). Even though we do not have access to the binding kinetics data for most of the antibodies in this assessment, AbC459 had the highest on-rate among the AbCellera antibodies available (data not shown) and proved optimal for the LFA format but not for the liquid immunoassay. Another possibility is the source of the trimeric spike protein preparation used in liquid and LF assays. LFA used materials procured from Sino Biological and produced in-house, while the liquid immunoassay exclusively used S protein procured from Acro Biosystems.

Our goal is to qualify reagents and methods that are publicly available to any developer who sees value in their use, removing the need for them to invest time and resources on antibodies with little or no potential. Further work is ongoing with our groups to develop a POC LFA with the potential for manufacturing at scale. An advantage of using recombinant antibodies like those from AbCellera and Leinco is that the variable antibody region of single antigen-specific memory B cells derived from convalescent patients is cloned into an expression vector, enabling cost-efficient scaled production of antibodies. In addition, this work uses recombinant IgG antibodies which are monomeric, with the possibility of manipulating the same variable region sequences to create recombinant IgM type antibodies, decameric forms of which may improve capture and/or detector efficiency leading to more effective rapid antigen assays for COVID-19 diagnosis.

## 4 Materials and Methods

### 4.1 Antibodies and Antigens

Commercially available antibodies to the S glycoprotein were procured from Leinco Technologies (Fenton, MO), Sino Biological (Wayne, PA), Cedar Lane (Burlington, NC), and Creative Diagnostics (Shirley, NY). AbCellera Biologics Inc. (Vancouver, BC, Canada) provided 41 recombinant antibodies engineered from B cells harvested from a convalescent patient after SARS-CoV-2 infection. A list of all anti-S antibodies screened in this work, their target region, and other relevant information are provided in Table S1 (supporting information).

The full-length trimeric SARS-CoV-2 S antigens expressed in mammalian cells were purchased from Acro Biosystems (Newark, DE) and Leinco Technologies or made in-house (Global Health Labs) using vector NR-52394 from Biodefense and Emerging Infections Research Resources Repository (BEI Resources, Manassas, VA). S antigens expressed in Baculovirus-insect cells were obtained from Sino Biological and BEI Resources. Heat-inactivated and γ-irradiated cells of MERS and SARS-CoV were also acquired from BEI Resources. Titered HEK293 cell culture supernatants of human coronaviruses OC43 and 229E were generously gifted from the laboratory of Dr. Scott Meschke, University of Washington (Seattle, WA). SARS-CoV-2 positive and negative nasopharyngeal specimens were obtained from the Washington COVID-19 Biorepository.^34^ These samples were discarded clinical specimen from a laboratory that used the Applied Biosystems TaqPath COVID-19 assay (ThermoFisher Scientific, Waltham, MA), a SARS-CoV-2 RT-PCR assay with FDA EUA.

### 4.2 Viral Load Determination via qRT-PCR

Clinical specimens were prepared in one of two ways. (1) RNA was extracted from 50 μL of specimen using the QIAamp Viral RNA Mini Kit (Qiagen, Valencia) according to the manufacturer’s instructions (2) 40 μL of specimen were heated to 95 °C for 10 min to lyse virions. Next, 5 μL of extracted RNA or 2.5 μL of heat-treated specimens were added to qRT-PCR reactions containing TaqPath 1-Step RT-qPCR Master Mix (ThermoFisher Scientific) and the U.S. Centers for Disease Control (CDC) and Prevention N1 primer set (IDT, Coralville). Reactions were carried out per the CDC protocol with an ABI7300 fast real-time PCR system (Applied Biosystems). A standard curve was generated using quantified genomic RNA from SARS-related coronavirus-2, isolate USA-WA1/2020, NR-52285 (BEI Resources), and used to determine the viral load of each sample.

### 4.3 Liquid Immunoassay Screening for Optimal Antibody Pairs

Sandwich ECL immunoassays^35^ were carried out using assay kits, instrumentation, and multiwell U-Plex plate consumables from MSD. The MSD U-Plex plates have integrated screen-printed carbon ink electrodes at the bottom of each well that are used as solid-phase supports for binding antibody–antigen reactions and as the source of electrical energy for inducing ECL from ECL labels in the detector antibodies.

Per the protocol, two aliquots of each antibody (100 μg/mL in PBS) were labeled with biotin (EZ-Link Sulfo-NHS-LC-biotinylation kit, ThermoFisher Scientific) for capture and SULFO-TAG (GOLD SULFO-TAG NHS-Ester, MSD, Rockville, MD) for detection. Unbound biotin or SULFO-TAG was removed using Zeba spin desalting columns (ThermoFisher Scientific), and the incorporation ratio for each label was measured. Briefly, the concentration of biotinylated antibodies after desalting was measured at 280 nm via a spectrophotometer (Nanodrop ND-1000, ThermoFisher Scientific); biotin incorporation was measured using a biotin quantification kit (Pierce, ThermoFisher Scientific) (Table S10). For measuring the incorporation of SULFO-TAG, the protein concentration was estimated using the bicinchoninic acid protein assay (ThermoFisher Scientific), and the SULFO-TAG label spectrophotometrically measured at 455 nm (Table S11).

The biotinylated capture antibodies were coupled to U-PLEX plates via biotin-streptavidin binding to U-PLEX linkers. To prepare the capture antibody arrays, up to 10 antibody-linker conjugates were combined in U-PLEX stop buffer at a concentration of 0.29 μg/mL per antibody, and 50 μL of this mixture was added to individual wells of the plates. The plates were incubated for 1 h with shaking (500 rpm) to allow the antibody array to self-assemble to the complimentary antibody-linker binding sites, and unbound material then removed by washing three times with 150 μL/well of 1× phosphate-buffered saline + 0.05% Tween-20 (PBS-T, pH 7.5) using a BioTek ELX405R microplate washer (BioTek Instruments Inc., Winooski, VT).

Appropriate serial dilutions of the trimeric S glycoprotein were prepared in Diluent 100 (MSD). Clinical specimens and cell lysates were diluted 1:1 in Diluent 100, and 50 μL of each diluted sample was added to each antibody array well in the plate and incubated with shaking for 1 h at room temperature. Plates were washed three times with PBS-T and then 25 μL of 2 μg/mL SULFO-TAG-labeled detection antibody in Diluent 3 (MSD) was added to each well, and then incubated for an hour with shaking. Plates were then washed three times to remove excess detection reagent and the wells filled with 150 μL of 2× read buffer T (MSD). The plates were inserted into the MESO QuickPlex SQ 120 plate reader (MSD), and the ECL from each individual array spot was subsequently measured. In the absence of a control, the array spot that gave the highest S/N in each plate was expressed as 100%, and each of the array spots in each plate expressed as percentile of this value. When serial dilutions of the S glycoprotein were used to generate a calibration curve, the relationship of ECL to S glycoprotein concentration was fitted to a four-parameter logistic (4-PL) function in the Discovery Workbench v4 program. The LOD was calculated from the fitted curve. S glycoprotein concentrations for γ-irradiated SARS-CoV-2 were calculated by back-fitting ECL to the 4-PL fit.

Antibodies were screened in a matrix format, acting both as capture and detector antibody. The identification of optimal antibody pairs for capture and detection of the S glycoprotein was determined via a three-stage process.

Round 1. All 41 AbCellera antibodies were screened in a matrix format using 10 ng/mL of trimeric S antigen (Acro Biosystems) in duplicate. The capture and detection antibody pairs that recorded 25% or greater ECL per plate were evaluated further over a greater range of S antigen concentrations (1000, 100, and 10 ng/mL) to verify the initial results. The highest ECL readings across each concentration ranges were then used to rank antibodies for round 2 screening.

Round 2. Six antibody candidates from round 1 were evaluated further alongside three antibodies from Sino Biological using seven-point dilutions of the S glycoprotein antigen in Diluent 100 (ranging from 1250 to 0.016 pg/mL) in duplicate. Antibody pairs were ranked in terms of the LOD.

Round 3: An additional four antibodies from Leinco were evaluated with the four best-performing antibodies from round 2, and 2 from round 1. The analytical sensitivity of the antibody pairs was determined from a seven-point calibration curve of the S antigen, and from a dilution series of the irradiated SARS-CoV-2. Specificity was evaluated by challenging the pairs with irradiated viral cultures and supernatants of other human coronavirus species (OC43, 229E, MERS, and SARS) at concentrations equivalent to 10^4^ TCID_50_/mL or PFU/mL in Diluent 100. Antibody pairs were ranked in terms of LOD and ECL signal with the best-performing pair further evaluated for clinical sensitivity and specificity with 53 clinical specimens.

### 4.4 Lateral Flow Assay Screening for Optimal Antibody Pairs

Concentration was measured for all proteins using bicinchoninic acid assay (ThermoFisher Scientific). Antigens were evaluated for purity and size using SDS-PAGE. Samples were premixed with NuPAGE LDS sample buffer (ThermoFisher Scientific), heated to 70 °C for 10 min, and ran on a 4–12% Bis-Tris gradient gel, with Novex Sharp prestained protein standard (ThermoFisher Scientific) as marker. Gel was stained using Coomassie Imperial Protein Stain (ThermoFisher Scientific) to visualize protein bands.

Latex beads were prepared as follows: For both test and control line detection conjugates, 400 nm carboxylic blue latex beads (CAB400NM, Magsphere, Pasadena CA) were washed three times with 0.1 M MES buffer (pH 6) and then activated using EDC/NHS (ThermoFisher Scientific) coupling reagents at 0.15 and 10 mg/mL, respectively, for 30 min. Afterward, the blue latex beads were conjugated in 1× PBS (pH 7.2) to various antispike antibodies at a bead: antibody (w/w) ratio of 20:1 and 10:1 for test and control line antibodies, respectively, for 3 h. Finally, latex conjugates were quenched using 0.1 M ethanolamine, washed, and blocked overnight with 6% (w/v) casein in water. The latex conjugates were stored in buffer containing 50 mM borate (pH 8.5) and 1% casein. The latex conjugates were quantified by measuring absorbance at 660 nm and comparing to absorbance of unconjugated beads.

For LFA reagent deposition and strip assembly, unlabeled capture antibodies were diluted to 1 mg/mL in PBS with 2.5% (w/v) sucrose and were stripped on 20 mm wide nitrocellulose CN95 (Sartorius Lab Instruments, Göttingen, Germany) (test line) at 1 μL/cm using ZX1010 dispense platform (BioDot, Irvine, CA) and dried at 25 °C for 30 min. The control line was striped with 0.75 mg/mL donkey antichicken IgY (Jackson ImmunoResearch, West Grove, PA) at 1 μL/cm. The test and control lines were striped at 8 and 13 mm from the upstream edge of the nitrocellulose membrane, respectively. For antibody screening, nitrocellulose was left unblocked. The conjugate pad (Grade 6613, Ahlstrom-Munksjö, Helsinki, Finland) was coated with two blocking solutions: 0.05% (w/v) Tween-20 in distilled water for 15–20 s and dried at 40 °C for 60 min, followed by 50 mM borate, pH 8.5 containing 0.25% (w/v) Triton X-100, 1% (w/v) Surfactant-10G, 1% (w/v) sucrose, and 6% (w/v) casein for 15–20 s. The conjugate pad was dried for 60 min at 40 °C before assembly. Card assembly was performed on a Matrix 2210 clamshell laminator (Kinematic Automation, Sonora, CA). Pads were placed on the backing card in the following order: nitrocellulose, cover tape, conjugate pad, sample pad, and wicking pad. Individual strips (3.3 mm wide) were cut with a Matrix 2360 sheet cutter (Kinematic Automation) and assembled in proprietary-designed cassettes using an assembly roller YK725 (Kinbio Tech Co., Shanghai, China).

Antibody pairs were screened on an integrated robotic system.^27,29^ In this system, the Hamilton STAR automated liquid handling robot (Hamilton Company, Reno, NV), a camera (UI-1460SE-C-HQ detector with a Tamron M118FM16 lens, IDS, Stoneham, MA), custom LFA holders, and custom control software developed at Global Health Labs were combined to allow rapid screening of antibody pairs directly in LFA format. The robot used eight-channel pipetting for parallel application to LFAs and a camera for imaging. The custom LFA framework held a maximum of 96 LFA cassettes per robot run. The custom control software applied 1 μL of latex bead conjugate mix (0.15% anti-S latex bead, 0.1 or 0.05% chicken IgY latex bead in 50 mM borate pH 8.5) to the conjugate pad in the LFA. After a 10 min delay to dry the conjugate mix, 75 μL of sample diluted in 2.5% BSA in PBS-T, S glycoprotein, or buffer (2.5% BSA in PBS-T or 2.5% BSA and 1% IGEPAL in PBS) was added to the sample pad. Images were obtained 20 min after sample addition. Four technical replicates were run for each antibody pair per sample type. Recombinant S glycoprotein was used as antigen at 80 ng/mL. The initial LFA screen (round 1) used S glycoprotein from Sino Biological, which was the most accessible at that time. Succeeding runs (round 2) used a recombinant S antigen prepared at Global Health Labs using vector NR-52394 that was subsequently determined preferable. A complete list of all pairs screened from all rounds is in Table S1 (supporting information).

## Supporting Information Available

Figure S1, detection of γ-irradiated SARS-CoV-2 by the best-performing AbCellera antibody pairs; Table S1, a list of the antibodies described in this work; Table S2, antibody pairs targeting the spike protein; Table S3, epitope mapping of AbCellera antibodies; Table S4, the data used in Figure 1 to highlight the performance of the AbC525/AbC397 antibody pair on three different sources of spike protein; Table S5, the top 10 performing antibody pairs from AbCellera screened on the MSD platform; Table S6, the data from screening the top AbCellera candidates with Sino Biological antibodies; Table S7, the data from AbCellera antibodies used to generate Figure 2; Table S8, the data used to generate Figure 3; Table S9, the data used to generate Table 3; Table S10, the biotinylation efficiencies for each antibody used as a capture reagent in the MSD assay; and Table S11, the conjugation efficiency of the SULPHO-TAG for each antibody used as the detector reagent in the MSD assay (PDF)

## Notes

Other than funding the PATH authors’ salaries, the Bill & Melinda Gates Foundation did not have any additional role in the study design, data collection and analysis, decision to publish, or preparation of the manuscript. This research was, in part, funded by the U.S. Government. The views and conclusions contained in this document are those of the authors and should not be interpreted as representing the official policies, either expressed or implied, of the U.S. Government.
