# Supplementary material for: Screening Antibodies Raised against the Spike Glycoprotein of SARS-CoV-2 to Support the Development of Rapid Antigen Assays
Source: ACS Omega. 2021 Jul 27;6(31):20139–48. doi: 10.1021/acsomega.1c01321 (PMC8340086; doi:10.1021/acsomega.1c01321)
Supplement: Supplementary file 1 — ao1c01321_si_001.pdf [file ao1c01321_si_001.pdf]

Supporting information

Title: Screening antibodies raised against the spike glycoprotein of SARS-CoV-2 to support the development of rapid antigen assays

Authors: Jason L. Cantera[1‡], David M. Cate[2‡], Allison Golden[1], Roger B. Peck[1], Lorraine L. Lillis[1], Gonzalo J. Domingo[1], Eileen Murphy[1], Bryan C. Barnhart[3], Caitlin A. Anderson[2], Luis F. Alonzo[2], Veronika Glukhova[2], Gleda Hermansky[2], Brianda Barrios-Lopez[2], Ethan Spencer[2], Samantha Kuhn[2], Zeba Islam[4], Benjamin D. Grant[2], Lucas Kraft[3,] Karine Herve[3], Valentine de Puyraimond[3], Yuri Hwang[3], Puneet K. Dewan[2], Bernhard H. Weigl[2], Kevin P. Nichols[2], David S. Boyle[1*]

Affiliations: [1] PATH, 2201 Westlake Avenue, Suite 200, Seattle, WA 98121, USA

[2] Global Health Laboratories, 14360 SE Eastgate Way, Bellevue, WA 98007, USA

[3] AbCellera Biologics Inc., 2215 Yukon St, Vancouver, BC V5Y 0A1, Canada

[4] Intellectual Ventures Lab, 14360 SE Eastgate Way, Bellevue, WA 98007, USA

[‡] Both authors contributed equally to this work.

Contents:

Figure S1. Detection of gamma-irradiated SARS-CoV-2 (BEI Resource cat. No. NR-52287) by the best performing AbCellera antibody pairs. Virions were detected at 1.40E+03 TCID50/mL.

Table S1. Vendor and other data for the anti-spike glycoprotein antibodies screened via liquid and/or lateral flow immunoassay formats.

Table S2. Antibody pairs targeting the spike protein screened by LFA.

Table S3. Reactivity of the 41 AbCellera antibodies to four different sources of spike protein.

Table S4. Data used to generate Figure 1, demonstrating performance of 3 commercially available trimeric S glycoproteins screened via AbC525-AbC397 antibody pair.

Table S5. ECL signal and limit of detection (LOD) for the top ten performing AbC antibody pairs using trimeric S protein from Acro Biosystems. Antibody pairs are sorted by decreasing LOD.

Table S6. The ECL signals from the top antibody candidates in round 1 paired with 3 antibodies from Sino Biologicals.

Table S7. The ECL signals of AbCellera antibody pairs using 1000, 100, and 10 ng/mL of trimeric S protein antigen from Acros Biosystems. Antibody pairs are sorted at decreasing ECL signal at 100 ng/mL.

Table S8. The ECL from the round 3 top antibody pairs and detection of SARS-CoV-2 virions.

Table S9. Data for Table 3.

Table S10. Biotinylation efficiencies for each antibody used as a capture reagent in liquid immunoassay.

Table S11. The conjugation efficiency of the SULFO-TAG for each antibody used as the detector reagent in the liquid immunoassay.

Figure S1. Detection of gamma-irradiated SARS-CoV-2 (BEI Resource cat. No. NR-52287) by the best performing AbCellera antibody pairs. Virions were detected at 1.40E+03 TCID50/mL.

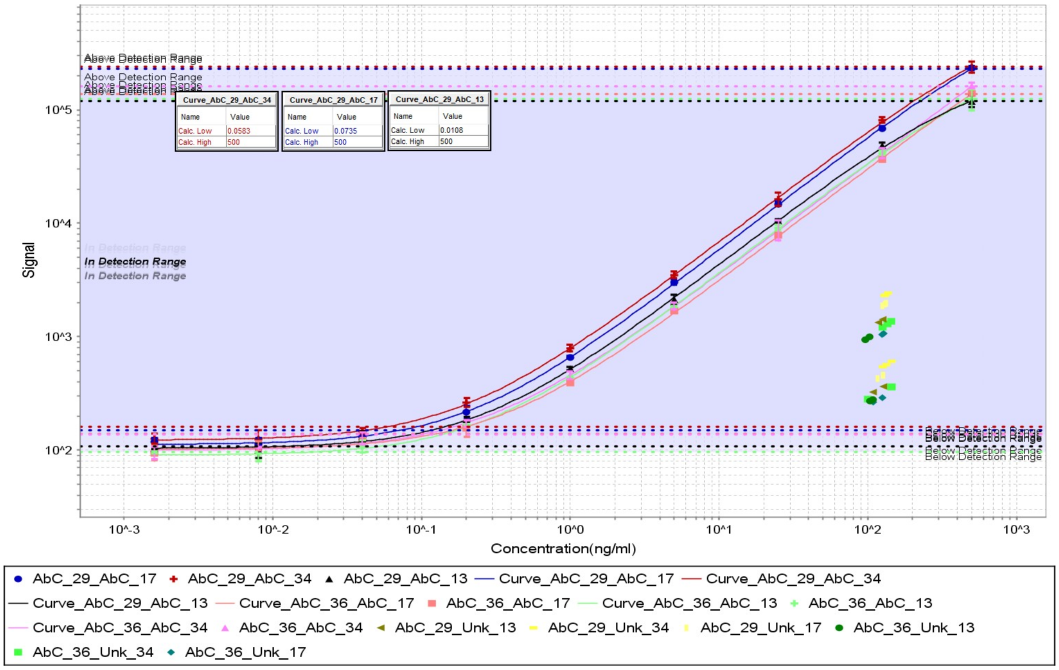

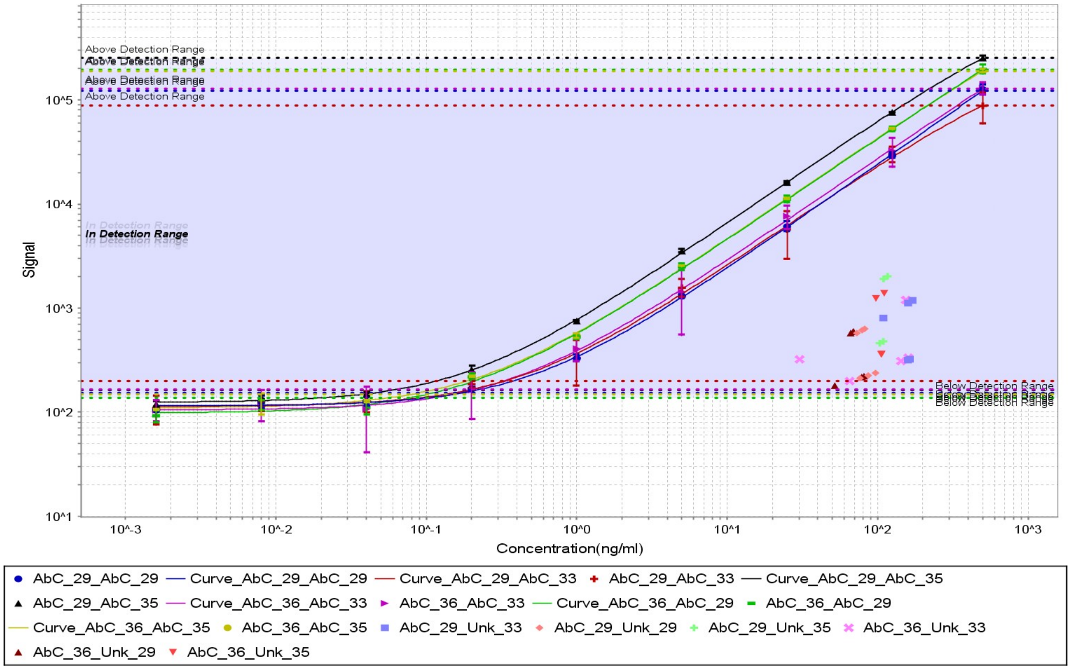

Table S1. Vendor and other data for the anti-spike glycoprotein antibodies screened via liquid and/or lateral flow immunoassay formats.

Vendor	Antibody name	Clone	Target region	Catalog / reference no.	Host	Isotype	Immunogen	Liquid immunoassay tested?	Lateral flow assay tested?
AbCellera	AbC275	275	S2	63974.1.a	Humanized	IgG1	Trimeric spike	Y	N
AbCellera	AbC277	277	S2	63997.1.a	Humanized	IgG1	Trimeric spike	Y	Y
AbCellera	AbC283	283	S2	63980.1.a	Humanized	IgG1	Trimeric spike	Y	Y
AbCellera	AbC285	285	S1	63983.1.a	Humanized	IgG1	Trimeric spike	Y	N
AbCellera	AbC291	291	S2	63986.1.a	Humanized	IgG1	Trimeric spike	Y	Y
AbCellera	AbC308	308	S2	63992.1.a	Humanized	IgG1	Trimeric spike	Y	Y
AbCellera	AbC310	310	undetermined	63997.1.a	Humanized	IgG1	Trimeric spike	Y	Y
AbCellera	AbC313	313	S2	64000.1.a	Humanized	IgG1	Trimeric spike	Y	Y
AbCellera	AbC353	353	S2	64003.1.a	Humanized	IgG1	Trimeric spike	Y	Y
AbCellera	AbC357	357	undetermined	64006.1.a	Humanized	IgG1	Trimeric spike	Y	N
AbCellera	AbC359	359	undetermined	64009.1.a	Humanized	IgG1	Trimeric spike	Y	N
AbCellera	AbC369	369	S2	64012.1.a		IgG1	Trimeric spike	Y	N
AbCellera	AbC397	397	S2	64020.1.a	Humanized	IgG1	Trimeric spike	Y	Y
AbCellera	AbC415	415	S2	64026.1.a	Humanized	IgG1	Trimeric spike	Y	N
AbCellera	AbC429	429	S2	64031.1.a	Humanized	IgG1	Trimeric spike	Y	Y
AbCellera	AbC453	453	S2	64036.1.a	Humanized	IgG1	Trimeric spike	Y	N
AbCellera	AbC459	459	S2	64042.1.a	Humanized	IgG1	Trimeric spike	Y	N
AbCellera	AbC463	463	S2	64045.1.a	Humanized	IgG1	Trimeric spike	Y	N
AbCellera	AbC478	478	undetermined	64051.1.a	Humanized	IgG1	Trimeric spike	Y	N
AbCellera	AbC489	489	S2	61061.1.a	Humanized	IgG1	Trimeric spike	Y	N
AbCellera	AbC491	491	undetermined	64064.1.a	Humanized	IgG1	Trimeric spike	Y	N
AbCellera	AbC500	500	S2	64067.1.a	Humanized	IgG1	Trimeric spike	Y	N
AbCellera	AbC530	530	undetermined	64082.1.a	Humanized	IgG1	Trimeric spike	Y	N
AbCellera	AbC554	554	S2	64087.1.a	Humanized	IgG1	Trimeric spike	Y	N
AbCellera	AbC258	258	S2	63971.1.a	Humanized	IgG1	Trimeric spike	Y	N
AbCellera	AbC298	298	S1	63989.1.a	Humanized	IgG1	Trimeric spike	Y	N
AbCellera	AbC393	393	RBD	64017.1.a	Humanized	IgG1	Trimeric spike	Y	N
AbCellera	AbC400	400	RBD	64023.1.a	Humanized	IgG1	Trimeric spike	Y	N
AbCellera	AbC447	447	RBD	63900.1.a	Humanized	IgG1	Trimeric spike	Y	N
AbCellera	AbC455	455	S2	64039.1.a	Humanized	IgG1	Trimeric spike	Y	N
AbCellera	AbC469	469	undetermined	64048.1.a	Humanized	IgG1	Trimeric spike	Y	N
AbCellera	AbC486	486	S2	64058.1.a	Humanized	IgG1	Trimeric spike	Y	N
AbCellera	AbC511	511	S2	64070.1.a	Humanized	IgG1	Trimeric spike	Y	N
AbCellera	AbC513	513	S2	64073.1.a	Humanized	IgG1	Trimeric spike	Y	N
AbCellera	AbC518	518	S2	64076.1.a	Humanized	IgG1	Trimeric spike	Y	N
AbCellera	AbC525	525	S2	64079.1.a	Humanized	IgG1	Trimeric spike	Y	Y
AbCellera	AbC557	557	S2	64090.1.a	Humanized	IgG1	Trimeric spike	Y	N
AbCellera	AbC558	558	S2	64093.1.a	Humanized	IgG1	Trimeric spike	Y	N
AbCellera	AbC561	561	S2	64096.1.a	Humanized	IgG1	Trimeric spike	Y	N
AbCellera	AbC574	574	undetermined	64101.1.a	Humanized	IgG1	Trimeric spike	Y	N
AbCellera	AbC585	585	S2	64104.1.a	Humanized	IgG1	Trimeric spike	Y	N
Creative Diagno	BIB112	BIB112	S1	CABT-CS031	Humanized	IgG	S1	N	Y
Creative Diagno	BIB112	BIB114	S1	CABT-CS033	Humanized	IgG	S1	N	Y
Sino Biological	D001	D001	undetermined	40150-D001	Mouse	IgG1	RBD	N	Y
Sino Biological	D002	D002	undetermined	40150-D002	Mouse	IgG1	RBD	N	Y
Sino Biological	D004	D004	undetermined	40150-D004	Mouse	IgG1	RBD	N	Y
Sino Biological	R007	R007	undetermined	40150-R007	Rabbit	IgG	Trimeric spike	N	Y
Sino Biological	D003	D003	RBD	40150-D003	Mouse/ human	IgG1	RBD	Y	N
Sino Biological	MM43	43	RBD	40591-MM43	Mouse	IgG1	S1	Y	Y
Sino Biological	MM57	57	RBD	40592-MM57	Mouse	IgG2b	RBD	Y	Y
Leinco	L2381	2381	RBD	LT4000	Human	IgG1	Trimeric spike	Y	Y

Leinco	L2838	2838	RBD	LT3000	Human	IgG1	Trimeric spike	Y	Y
Leinco	L2355	2355	RBD	LT5000	Human	IgG1	Trimeric spike	Y	Y
Leinco	L2215	2215	S1 NTD	LT6000	Human	IgG1	Trimeric spike	Y	Y
Leinco	L2136	2136	undetermined		Human	IgG1	Trimeric spike	N	Y
Leinco	L2143	2143	undetermined		Human	IgG1	Trimeric spike	N	Y
Leinco	L2146	2146	S1 NTD	LT2000	Human	IgG1	Trimeric spike	N	Y
Leinco	L2197	2197	S1 NTD		Human	IgG1	Trimeric spike	N	Y

Table S2. Antibody pairs targeting the spike protein screened by LFA.

Index	Capture antibody	Detector antibody	Average rank	
			round 1	round 2
**1**	AbC131	AbC131	39	-
**2**	AbC131	CD CABT-CS031[BIB112]	45	-
**3**	AbC131	CD CABT-CS033[BIB114]	54.5	-
**4**	AbC131	SB 40150-D001	25	-
**5**	AbC131	SB 40150-D002	36.5	-
**6**	AbC131	SB 40150-D003	50	-
**7**	AbC131	SB 40150-D004	46	-
**8**	AbC131	SB 40150-R007	35.5	-
**9**	AbC277	AbC277	-	551
**10**	AbC277	AbC283	-	349
**11**	AbC277	AbC291	-	473
**12**	AbC277	AbC308	-	113
**13**	AbC277	AbC310	-	440.5
**14**	AbC277	AbC313	-	368
**15**	AbC277	AbC353	-	563
**16**	AbC277	AbC397	-	549
**17**	AbC277	AbC429	-	222
**18**	AbC277	AbC447	-	139
**19**	AbC277	AbC459	-	392
**20**	AbC277	AbC525	-	523
**21**	AbC277	L2136	-	526.5
**22**	AbC277	L2143	-	149
**23**	AbC277	L2146	-	449
**24**	AbC277	L2197	-	308.5
**25**	AbC277	L2215	-	127.5
**26**	AbC277	L2355	-	13
**27**	AbC277	L2381	-	57
**28**	AbC277	L2838	-	106
**29**	AbC277	SB 40150-D001	-	25
**30**	AbC277	SB 40150-D002	-	17.5
**31**	AbC277	SB 40150-D004	-	113.5
**32**	AbC283	AbC277	-	433
**33**	AbC283	AbC283	-	551.5
**34**	AbC283	AbC291	-	390
**35**	AbC283	AbC308	-	309.5
**36**	AbC283	AbC310	-	463
**37**	AbC283	AbC313	-	321.5
**38**	AbC283	AbC353	-	458
**39**	AbC283	AbC397	-	548.5
**40**	AbC283	AbC429	-	302.5
**41**	AbC283	AbC447	-	300
**42**	AbC283	AbC459	-	349.5
**43**	AbC283	AbC525	-	548
**44**	AbC283	L2136	-	292.5

**45**	AbC283	L2143	-	247
**46**	AbC283	L2146	-	326.5
**47**	AbC283	L2197	-	382.5
**48**	AbC283	L2215	-	289
**49**	AbC283	L2355	-	76
**50**	AbC283	L2381	-	148.5
**51**	AbC283	L2838	-	120.5
**52**	AbC283	SB 40150-D001	-	114.5
**53**	AbC283	SB 40150-D002	-	156
**54**	AbC283	SB 40150-D004	-	223
**55**	AbC291	AbC277	-	381
**56**	AbC291	AbC283	-	451.5
**57**	AbC291	AbC291	-	482.5
**58**	AbC291	AbC308	-	244.5
**59**	AbC291	AbC310	-	332
**60**	AbC291	AbC313	-	523
**61**	AbC291	AbC353	-	498
**62**	AbC291	AbC397	-	525.5
**63**	AbC291	AbC429	-	214
**64**	AbC291	AbC447	-	101.5
**65**	AbC291	AbC459	-	443
**66**	AbC291	AbC525	-	433
**67**	AbC291	L2136	-	273.5
**68**	AbC291	L2143	-	210.5
**69**	AbC291	L2146	-	247
**70**	AbC291	L2197	-	224
**71**	AbC291	L2215	-	134.5
**72**	AbC291	L2355	-	16
**73**	AbC291	L2381	-	39.5
**74**	AbC291	L2838	-	40
**75**	AbC291	SB 40150-D001	-	64.5
**76**	AbC291	SB 40150-D002	-	43.5
**77**	AbC291	SB 40150-D004	-	65.5
**78**	AbC308	AbC277	-	510.5
**79**	AbC308	AbC283	-	497.5
**80**	AbC308	AbC291	-	386
**81**	AbC308	AbC308	-	480.5
**82**	AbC308	AbC310	-	518
**83**	AbC308	AbC313	-	319.5
**84**	AbC308	AbC353	-	284.5
**85**	AbC308	AbC397	-	550
**86**	AbC308	AbC429	-	451.5
**87**	AbC308	AbC447	-	262.5
**88**	AbC308	AbC459	-	199
**89**	AbC308	AbC525	-	354.5
**90**	AbC308	L2136	-	360.5
**91**	AbC308	L2143	-	183
**92**	AbC308	L2146	-	387
**93**	AbC308	L2197	-	226

**94**	AbC308	L2215	-	230.5
**95**	AbC308	L2355	-	99.5
**96**	AbC308	L2381	-	153
**97**	AbC308	L2838	-	78.5
**98**	AbC308	SB 40150-D001	-	206.5
**99**	AbC308	SB 40150-D002	-	157
**100**	AbC308	SB 40150-D004	-	129.5
**101**	AbC310	AbC277	-	430
**102**	AbC310	AbC283	-	534
**103**	AbC310	AbC291	-	302
**104**	AbC310	AbC308	-	336
**105**	AbC310	AbC310	-	546.5
**106**	AbC310	AbC313	-	317.5
**107**	AbC310	AbC353	-	338
**108**	AbC310	AbC397	-	464.5
**109**	AbC310	AbC429	-	267
**110**	AbC310	AbC447	-	246
**111**	AbC310	AbC459	-	256.5
**112**	AbC310	AbC525	-	408.5
**113**	AbC310	L2136	-	357.5
**114**	AbC310	L2143	-	290
**115**	AbC310	L2146	-	270.5
**116**	AbC310	L2197	-	336
**117**	AbC310	L2215	-	185.5
**118**	AbC310	L2355	-	99
**119**	AbC310	L2381	-	133
**120**	AbC310	L2838	-	45.5
**121**	AbC310	SB 40150-D001	-	121.5
**122**	AbC310	SB 40150-D002	-	379.5
**123**	AbC310	SB 40150-D004	-	231.5
**124**	AbC313	AbC277	-	470
**125**	AbC313	AbC283	-	446
**126**	AbC313	AbC291	-	473
**127**	AbC313	AbC308	-	293
**128**	AbC313	AbC310	-	339.5
**129**	AbC313	AbC313	-	452.5
**130**	AbC313	AbC353	-	536
**131**	AbC313	AbC397	-	503.5
**132**	AbC313	AbC429	-	303
**133**	AbC313	AbC447	-	227.5
**134**	AbC313	AbC459	-	533.5
**135**	AbC313	AbC525	-	391.5
**136**	AbC313	L2136	-	276
**137**	AbC313	L2143	-	239.5
**138**	AbC313	L2146	-	348.5
**139**	AbC313	L2197	-	344
**140**	AbC313	L2215	-	152.5
**141**	AbC313	L2355	-	47.5
**142**	AbC313	L2381	-	78

**143**	AbC313	L2838	-	25
**144**	AbC313	SB 40150-D001	-	63
**145**	AbC313	SB 40150-D002	-	68.5
**146**	AbC313	SB 40150-D004	-	165
**147**	AbC353	AbC277	-	559
**148**	AbC353	AbC283	-	415.5
**149**	AbC353	AbC291	-	485
**150**	AbC353	AbC308	-	268
**151**	AbC353	AbC310	-	421
**152**	AbC353	AbC313	-	451.5
**153**	AbC353	AbC353	-	507
**154**	AbC353	AbC397	-	464
**155**	AbC353	AbC429	-	429.5
**156**	AbC353	AbC447	-	143
**157**	AbC353	AbC459	-	551.5
**158**	AbC353	AbC525	-	520.5
**159**	AbC353	L2136	-	223
**160**	AbC353	L2143	-	247
**161**	AbC353	L2146	-	397.5
**162**	AbC353	L2197	-	329
**163**	AbC353	L2215	-	226
**164**	AbC353	L2355	-	66.5
**165**	AbC353	L2381	-	92.5
**166**	AbC353	L2838	-	133
**167**	AbC353	SB 40150-D001	-	50.5
**168**	AbC353	SB 40150-D002	-	88.5
**169**	AbC353	SB 40150-D004	-	170.5
**170**	AbC397	AbC277	-	458.5
**171**	AbC397	AbC283	-	513
**172**	AbC397	AbC291	-	342.5
**173**	AbC397	AbC308	-	432
**174**	AbC397	AbC310	-	376.5
**175**	AbC397	AbC313	-	390.5
**176**	AbC397	AbC353	-	400.5
**177**	AbC397	AbC397	-	502.5
**178**	AbC397	AbC429	-	439.5
**179**	AbC397	AbC447	-	351.5
**180**	AbC397	AbC459	-	249.5
**181**	AbC397	AbC525	-	541
**182**	AbC397	L2136	-	357.5
**183**	AbC397	L2143	-	402.5
**184**	AbC397	L2146	-	479
**185**	AbC397	L2197	-	525.5
**186**	AbC397	L2215	-	486
**187**	AbC397	L2355	-	270.5
**188**	AbC397	L2381	-	350.5
**189**	AbC397	L2838	-	297.5
**190**	AbC397	SB 40150-D001	-	288.5
**191**	AbC397	SB 40150-D002	-	530.5

**192**	AbC397	SB 40150-D004	-	455.5
**193**	AbC429	AbC277	-	415.5
**194**	AbC429	AbC283	-	493
**195**	AbC429	AbC291	-	174
**196**	AbC429	AbC308	-	413.5
**197**	AbC429	AbC310	-	444
**198**	AbC429	AbC313	-	193
**199**	AbC429	AbC353	-	335
**200**	AbC429	AbC397	-	545.5
**201**	AbC429	AbC429	-	422.5
**202**	AbC429	AbC447	-	236
**203**	AbC429	AbC459	-	128
**204**	AbC429	AbC525	-	226
**205**	AbC429	L2136	-	281
**206**	AbC429	L2143	-	163.5
**207**	AbC429	L2146	-	303
**208**	AbC429	L2197	-	252.5
**209**	AbC429	L2215	-	215.5
**210**	AbC429	L2355	-	48.5
**211**	AbC429	L2381	-	87
**212**	AbC429	L2838	-	99.5
**213**	AbC429	SB 40150-D001	-	43
**214**	AbC429	SB 40150-D002	-	63
**215**	AbC429	SB 40150-D004	-	149.5
**216**	AbC447	AbC277	-	331.5
**217**	AbC447	AbC283	-	334.5
**218**	AbC447	AbC291	-	95.5
**219**	AbC447	AbC308	-	147
**220**	AbC447	AbC310	-	329.5
**221**	AbC447	AbC313	-	80.5
**222**	AbC447	AbC353	-	192
**223**	AbC447	AbC397	-	428.5
**224**	AbC447	AbC429	-	102
**225**	AbC447	AbC447	-	552
**226**	AbC447	AbC459	-	53.5
**227**	AbC447	AbC525	-	188.5
**228**	AbC447	L2136	-	350.5
**229**	AbC447	L2143	-	107
**230**	AbC447	L2146	-	288.5
**231**	AbC447	L2165	-	542
**232**	AbC447	L2197	-	397
**233**	AbC447	L2215	-	134
**234**	AbC447	L2355	-	396.5
**235**	AbC447	L2381	-	471.5
**236**	AbC447	L2838	-	511.5
**237**	AbC447	SB 40150-D001	-	88
**238**	AbC447	SB 40150-D002	-	143.5
**239**	AbC447	SB 40150-D004	-	139.5
**240**	AbC447	SB 40591-MM43[43]	-	223

**241**	AbC459	AbC277	-	403
**242**	AbC459	AbC283	-	365.5
**243**	AbC459	AbC291	-	369
**244**	AbC459	AbC308	-	123.5
**245**	AbC459	AbC310	-	327.5
**246**	AbC459	AbC313	-	505.5
**247**	AbC459	AbC353	-	480
**248**	AbC459	AbC397	-	468.5
**249**	AbC459	AbC429	-	171
**250**	AbC459	AbC447	-	156.5
**251**	AbC459	AbC459	-	391.5
**252**	AbC459	AbC525	-	405
**253**	AbC459	L2136	-	159
**254**	AbC459	L2143	-	136.5
**255**	AbC459	L2146	-	207.5
**256**	AbC459	L2165	-	249.5
**257**	AbC459	L2197	-	332
**258**	AbC459	L2215	-	80
**259**	AbC459	L2355	-	1.5
**260**	AbC459	L2381	-	38.5
**261**	AbC459	L2838	-	47.5
**262**	AbC459	SB 40150-D001	-	1.5
**263**	AbC459	SB 40150-D002	-	29
**264**	AbC459	SB 40150-D004	-	121
**265**	AbC459	SB 40591-MM43[43]	-	95.5
**266**	AbC525	AbC277	-	487
**267**	AbC525	AbC283	-	499.5
**268**	AbC525	AbC291	-	545
**269**	AbC525	AbC308	-	156
**270**	AbC525	AbC310	-	312
**271**	AbC525	AbC313	-	383
**272**	AbC525	AbC353	-	549
**273**	AbC525	AbC397	-	526.5
**274**	AbC525	AbC429	-	174.5
**275**	AbC525	AbC447	-	227
**276**	AbC525	AbC459	-	229.5
**277**	AbC525	AbC525	-	515.5
**278**	AbC525	L2136	-	124
**279**	AbC525	L2143	-	137
**280**	AbC525	L2146	-	235.5
**281**	AbC525	L2165	-	474.5
**282**	AbC525	L2197	-	292.5
**283**	AbC525	L2215	-	132
**284**	AbC525	L2355	-	9
**285**	AbC525	L2381	-	75.5
**286**	AbC525	L2838	-	62.5
**287**	AbC525	SB 40150-D001	-	59
**288**	AbC525	SB 40150-D002	-	29.5
**289**	AbC525	SB 40150-D004	-	100

**290**	AbC525	SB 40591-MM43[43]	-	170.5
**291**	CABT-CS031[BIB1	AbC131	32.5	-
**292**	CABT-CS031[BIB1	CD CABT-CS031[BIB112]	58	-
**293**	CABT-CS031[BIB1	CD CABT-CS033[BIB114]	25	-
**294**	CABT-CS031[BIB1	SB 40150-D001	25	-
**295**	CABT-CS031[BIB1	SB 40150-D002	31	-
**296**	CABT-CS031[BIB1	SB 40150-D003	29.5	-
**297**	CABT-CS031[BIB1	SB 40150-D004	11	-
**298**	CABT-CS031[BIB1	SB 40150-R007	23	-
**299**	CABT-CS033[BIB1	AbC131	39.5	-
**300**	CABT-CS033[BIB1	CD CABT-CS031[BIB112]	20.5	-
**301**	CABT-CS033[BIB1	CD CABT-CS033[BIB114]	56	-
**302**	CABT-CS033[BIB1	SB 40150-D001	48	-
**303**	CABT-CS033[BIB1	SB 40150-D002	49	-
**304**	CABT-CS033[BIB1	SB 40150-D003	17	-
**305**	CABT-CS033[BIB1	SB 40150-D004	12	-
**306**	CABT-CS033[BIB1	SB 40150-R007	13	-
**307**	L2143	AbC277	-	536
**308**	L2143	AbC283	-	409
**309**	L2143	AbC291	-	201.5
**310**	L2143	AbC308	-	267
**311**	L2143	AbC310	-	562
**312**	L2143	AbC313	-	216.5
**313**	L2143	AbC353	-	552
**314**	L2143	AbC397	-	512
**315**	L2143	AbC429	-	317.5
**316**	L2143	AbC447	-	281.5
**317**	L2143	AbC459	-	147
**318**	L2143	AbC525	-	303
**319**	L2143	L2136	-	264
**320**	L2143	L2143	-	520.5
**321**	L2143	L2146	-	234
**322**	L2143	L2165	-	527
**323**	L2143	L2197	-	547.5
**324**	L2143	L2215	-	179.5
**325**	L2143	L2355	-	104.5
**326**	L2143	L2381	-	200.5
**327**	L2143	L2838	-	181
**328**	L2143	SB 40150-D001	-	273
**329**	L2143	SB 40150-D002	-	171
**330**	L2143	SB 40150-D004	-	379
**331**	L2146	AbC277	-	521.5
**332**	L2146	AbC283	-	456
**333**	L2146	AbC291	-	349
**334**	L2146	AbC308	-	262
**335**	L2146	AbC310	-	425.5
**336**	L2146	AbC313	-	331.5
**337**	L2146	AbC353	-	323.5
**338**	L2146	AbC397	-	437

**339**	L2146	AbC415	-	542.5
**340**	L2146	AbC429	-	306
**341**	L2146	AbC447	-	331
**342**	L2146	AbC459	-	105.5
**343**	L2146	AbC525	-	277.5
**344**	L2146	L2136	-	385
**345**	L2146	L2143	-	149
**346**	L2146	L2146	-	547
**347**	L2146	L2165	-	437.5
**348**	L2146	L2197	-	300
**349**	L2146	L2215	-	397
**350**	L2146	L2355	-	140.5
**351**	L2146	L2381	-	170.5
**352**	L2146	L2838	-	114.5
**353**	L2146	SB 40150-D001	-	384.5
**354**	L2146	SB 40150-D002	-	336
**355**	L2146	SB 40150-D004	-	194
**356**	L2146	SB 40591-MM43[43]	-	321
**357**	L2149	AbC277	-	407.5
**358**	L2149	AbC283	-	454.5
**359**	L2149	AbC291	-	475.5
**360**	L2149	AbC308	-	286
**361**	L2149	AbC310	-	537
**362**	L2149	AbC313	-	281.5
**363**	L2149	AbC353	-	547.5
**364**	L2149	AbC397	-	559.5
**365**	L2149	AbC429	-	373
**366**	L2149	AbC447	-	380
**367**	L2149	AbC459	-	230.5
**368**	L2149	AbC525	-	308.5
**369**	L2149	L2136	-	419.5
**370**	L2149	L2143	-	345.5
**371**	L2149	L2146	-	476.5
**372**	L2149	L2165	-	497
**373**	L2149	L2197	-	539
**374**	L2149	L2215	-	226.5
**375**	L2149	L2355	-	114
**376**	L2149	L2381	-	275.5
**377**	L2149	L2838	-	208.5
**378**	L2149	SB 40150-D001	-	258
**379**	L2149	SB 40150-D002	-	192.5
**380**	L2149	SB 40150-D004	-	217.5
**381**	L2165	AbC277	-	530
**382**	L2165	AbC283	-	380
**383**	L2165	AbC291	-	214
**384**	L2165	AbC308	-	222.5
**385**	L2165	AbC310	-	483
**386**	L2165	AbC313	-	176
**387**	L2165	AbC353	-	327.5

**388**	L2165	AbC397	-	477
**389**	L2165	AbC429	-	226.5
**390**	L2165	AbC447	-	524.5
**391**	L2165	AbC459	-	74.5
**392**	L2165	AbC525	-	279.5
**393**	L2165	L2136	-	462.5
**394**	L2165	L2143	-	255.5
**395**	L2165	L2146	-	280.5
**396**	L2165	L2165	-	404.5
**397**	L2165	L2197	-	551.5
**398**	L2165	L2215	-	281
**399**	L2165	L2355	-	529
**400**	L2165	L2381	-	500.5
**401**	L2165	L2838	-	537.5
**402**	L2165	SB 40150-D001	-	274
**403**	L2165	SB 40150-D002	-	230
**404**	L2165	SB 40150-D004	-	235.5
**405**	L2215	AbC277	-	285.5
**406**	L2215	AbC283	-	369.5
**407**	L2215	AbC291	-	140
**408**	L2215	AbC308	-	139
**409**	L2215	AbC310	-	361.5
**410**	L2215	AbC313	-	147.5
**411**	L2215	AbC353	-	388
**412**	L2215	AbC397	-	379
**413**	L2215	AbC415	-	322
**414**	L2215	AbC429	-	256
**415**	L2215	AbC447	-	235
**416**	L2215	AbC459	-	27
**417**	L2215	AbC525	-	300.5
**418**	L2215	L2136	-	433
**419**	L2215	L2143	-	133
**420**	L2215	L2146	-	462
**421**	L2215	L2197	-	428
**422**	L2215	L2215	-	484
**423**	L2215	L2355	-	92.5
**424**	L2215	L2381	-	107
**425**	L2215	L2838	-	70.5
**426**	L2215	SB 40150-D001	-	296
**427**	L2215	SB 40150-D002	-	321.5
**428**	L2215	SB 40150-D004	-	174.5
**429**	L2355	AbC277	-	210.5
**430**	L2355	AbC283	-	266
**431**	L2355	AbC291	-	35.5
**432**	L2355	AbC308	-	58.5
**433**	L2355	AbC310	-	210
**434**	L2355	AbC313	-	61.5
**435**	L2355	AbC353	-	454.5
**436**	L2355	AbC397	-	559.5

**437**	L2355	AbC415	-	364
**438**	L2355	AbC429	-	95
**439**	L2355	AbC447	-	437
**440**	L2355	AbC459	-	5.5
**441**	L2355	AbC525	-	104
**442**	L2355	L2136	-	201
**443**	L2355	L2143	-	55
**444**	L2355	L2146	-	233.5
**445**	L2355	L2197	-	162
**446**	L2355	L2215	-	175
**447**	L2355	L2355	-	304.5
**448**	L2355	L2381	-	315
**449**	L2355	L2838	-	429
**450**	L2355	SB 40150-D001	-	65.5
**451**	L2355	SB 40150-D002	-	78
**452**	L2355	SB 40150-D004	-	64.5
**453**	L2381	AbC277	-	347.5
**454**	L2381	AbC283	-	295
**455**	L2381	AbC291	-	119.5
**456**	L2381	AbC308	-	101.5
**457**	L2381	AbC310	-	311
**458**	L2381	AbC313	-	90.5
**459**	L2381	AbC353	-	329.5
**460**	L2381	AbC397	-	481.5
**461**	L2381	AbC415	-	208
**462**	L2381	AbC429	-	168
**463**	L2381	AbC447	-	502
**464**	L2381	AbC459	-	25.5
**465**	L2381	AbC525	-	217.5
**466**	L2381	L2136	-	219
**467**	L2381	L2143	-	94
**468**	L2381	L2146	-	255.5
**469**	L2381	L2165	-	429.5
**470**	L2381	L2197	-	205.5
**471**	L2381	L2215	-	274
**472**	L2381	L2355	-	339.5
**473**	L2381	L2381	-	428
**474**	L2381	L2838	-	445
**475**	L2381	SB 40150-D001	-	103
**476**	L2381	SB 40150-D002	-	190
**477**	L2381	SB 40150-D004	-	101.5
**478**	L2381	SB 40591-MM43[43]	-	118.5
**479**	L2838	AbC277	-	345
**480**	L2838	AbC283	-	257
**481**	L2838	AbC291	-	123
**482**	L2838	AbC308	-	61
**483**	L2838	AbC310	-	449.5
**484**	L2838	AbC313	-	70
**485**	L2838	AbC353	-	300.5

**486**	L2838	AbC397	-	497.5
**487**	L2838	AbC415	-	284
**488**	L2838	AbC429	-	166.5
**489**	L2838	AbC447	-	480
**490**	L2838	AbC459	-	20
**491**	L2838	AbC525	-	186.5
**492**	L2838	L2136	-	199.5
**493**	L2838	L2143	-	81.5
**494**	L2838	L2146	-	224
**495**	L2838	L2165	-	446
**496**	L2838	L2197	-	137
**497**	L2838	L2215	-	171.5
**498**	L2838	L2355	-	351.5
**499**	L2838	L2381	-	356.5
**500**	L2838	L2838	-	440
**501**	L2838	SB 40150-D001	-	69.5
**502**	L2838	SB 40150-D002	-	91
**503**	L2838	SB 40150-D004	-	98
**504**	L2838	SB 40591-MM43[43]	-	179.5
**505**	SB 40150-D001	AbC131	38	-
**506**	SB 40150-D001	CD CABT-CS031[BIB112]	43.5	-
**507**	SB 40150-D001	CD CABT-CS033[BIB114]	43	-
**508**	SB 40150-D001	SB 40150-D001	22.5	-
**509**	SB 40150-D001	SB 40150-D002	28	-
**510**	SB 40150-D001	SB 40150-D003	9	-
**511**	SB 40150-D001	SB 40150-D004	3	-
**512**	SB 40150-D001	SB 40150-R007	16.5	-
**513**	SB 40150-D002	AbC131	61	-
**514**	SB 40150-D002	AbC277	-	379.5
**515**	SB 40150-D002	AbC283	-	402
**516**	SB 40150-D002	AbC291	-	218
**517**	SB 40150-D002	AbC308	-	282.5
**518**	SB 40150-D002	AbC310	-	522
**519**	SB 40150-D002	AbC313	-	156
**520**	SB 40150-D002	AbC353	-	350
**521**	SB 40150-D002	AbC397	-	512.5
**522**	SB 40150-D002	AbC429	-	243.5
**523**	SB 40150-D002	AbC459	-	11
**524**	SB 40150-D002	AbC525	-	260.5
**525**	SB 40150-D002	CD CABT-CS031[BIB112]	53.5	-
**526**	SB 40150-D002	CD CABT-CS033[BIB114]	62.5	-
**527**	SB 40150-D002	L2146	-	358
**528**	SB 40150-D002	L2165	-	386
**529**	SB 40150-D002	L2197	-	276
**530**	SB 40150-D002	L2215	-	265
**531**	SB 40150-D002	L2355	-	67
**532**	SB 40150-D002	L2381	-	81.5
**533**	SB 40150-D002	L2838	-	126.5
**534**	SB 40150-D002	SB 40150-D001	32.5	420.5

**535**	SB 40150-D002	SB 40150-D002	21	394
**536**	SB 40150-D002	SB 40150-D003	10	-
**537**	SB 40150-D002	SB 40150-D004	6.5	147
**538**	SB 40150-D002	SB 40150-R007	35.5	-
**539**	SB 40150-D003	AbC131	52.5	-
**540**	SB 40150-D003	AbC277	-	316.5
**541**	SB 40150-D003	AbC283	-	345
**542**	SB 40150-D003	AbC291	-	146
**543**	SB 40150-D003	AbC308	-	249.5
**544**	SB 40150-D003	AbC310	-	448.5
**545**	SB 40150-D003	AbC313	-	241.5
**546**	SB 40150-D003	AbC353	-	456.5
**547**	SB 40150-D003	AbC397	-	505.5
**548**	SB 40150-D003	AbC415	-	447
**549**	SB 40150-D003	AbC429	-	253
**550**	SB 40150-D003	AbC447	-	293.5
**551**	SB 40150-D003	AbC459	-	109
**552**	SB 40150-D003	AbC525	-	378
**553**	SB 40150-D003	CD CABT-CS031[BIB112]	6.5	-
**554**	SB 40150-D003	CD CABT-CS033[BIB114]	47	-
**555**	SB 40150-D003	L2136	-	286.5
**556**	SB 40150-D003	L2143	-	191.5
**557**	SB 40150-D003	L2146	-	320.5
**558**	SB 40150-D003	L2197	-	366.5
**559**	SB 40150-D003	L2215	-	294
**560**	SB 40150-D003	L2355	-	120.5
**561**	SB 40150-D003	L2381	-	120.5
**562**	SB 40150-D003	L2838	-	169
**563**	SB 40150-D003	SB 40150-D001	4.5	156.5
**564**	SB 40150-D003	SB 40150-D002	1	302
**565**	SB 40150-D003	SB 40150-D003	34.5	-
**566**	SB 40150-D003	SB 40150-D004	15.5	408.5
**567**	SB 40150-D003	SB 40150-R007	60.5	-
**568**	SB 40150-D004	AbC131	42.5	-
**569**	SB 40150-D004	AbC277	-	187
**570**	SB 40150-D004	AbC283	-	191.5
**571**	SB 40150-D004	AbC291	-	71
**572**	SB 40150-D004	AbC308	-	64
**573**	SB 40150-D004	AbC310	-	331
**574**	SB 40150-D004	AbC313	-	55.5
**575**	SB 40150-D004	AbC353	-	247
**576**	SB 40150-D004	AbC397	-	510.5
**577**	SB 40150-D004	AbC429	-	95
**578**	SB 40150-D004	AbC459	-	26.5
**579**	SB 40150-D004	AbC525	-	184.5
**580**	SB 40150-D004	CD CABT-CS031[BIB112]	8	-
**581**	SB 40150-D004	CD CABT-CS033[BIB114]	51	-
**582**	SB 40150-D004	L2146	-	209.5
**583**	SB 40150-D004	L2197	-	93.5

**584**	SB 40150-D004	L2215	-	107
**585**	SB 40150-D004	L2355	-	39
**586**	SB 40150-D004	L2381	-	32
**587**	SB 40150-D004	L2838	-	42.5
**588**	SB 40150-D004	SB 40150-D001	4	-
**589**	SB 40150-D004	SB 40150-D002	2.5	-
**590**	SB 40150-D004	SB 40150-D003	63.5	-
**591**	SB 40150-D004	SB 40150-D004	15.5	289.5
**592**	SB 40150-D004	SB 40150-R007	24.5	-
**593**	SB 40591-MM43[43]	AbC277	-	381
**594**	SB 40591-MM43[43]	AbC283	-	445
**595**	SB 40591-MM43[43]	AbC291	-	105
**596**	SB 40591-MM43[43]	AbC308	-	81
**597**	SB 40591-MM43[43]	AbC310	-	510.5
**598**	SB 40591-MM43[43]	AbC313	-	57
**599**	SB 40591-MM43[43]	AbC353	-	470
**600**	SB 40591-MM43[43]	AbC397	-	548
**601**	SB 40591-MM43[43]	AbC429	-	153.5
**602**	SB 40591-MM43[43]	AbC447	-	278
**603**	SB 40591-MM43[43]	AbC459	-	51
**604**	SB 40591-MM43[43]	AbC525	-	173.5
**605**	SB 40591-MM43[43]	L2136	-	341.5
**606**	SB 40591-MM43[43]	L2143	-	27
**607**	SB 40591-MM43[43]	L2146	-	292
**608**	SB 40591-MM43[43]	L2197	-	230
**609**	SB 40591-MM43[43]	L2215	-	310.5
**610**	SB 40591-MM43[43]	L2355	-	11
**611**	SB 40591-MM43[43]	L2381	-	68
**612**	SB 40591-MM43[43]	L2838	-	53.5
**613**	SB 40591-MM43[43]	SB 40150-D004	-	76.5
**614**	SB 40150-R007	AbC131	53.5	-
**615**	SB 40150-R007	CD CABT-CS031[BIB112]	22	-
**616**	SB 40150-R007	CD CABT-CS033[BIB114]	59.5	-
**617**	SB 40150-R007	SB 40150-D001	58	-
**618**	SB 40150-R007	SB 40150-D002	40	-
**619**	SB 40150-R007	SB 40150-D003	26	-
**620**	SB 40150-R007	SB 40150-D004	19	-
**621**	SB 40150-R007	SB 40150-R007	61	-

Table S3. Reactivity of the 41 AbCellera antibodies to four different sources of spike protein.

	ECL signals using different S protein antigens			
**Antibodies**	**Trimeric S, Acro Biosystems SPN-C52H8**	**S1, Native Antigens, REC31806-500**	**RBD, Creative Diagnostics, DAGC089**	**S2, Native Antigens, REC31807-500**
AbC513	215121	146	197	4959
AbC447	164681	50475	216987	360
AbC459	153352	88	94	4731
AbC397	145149	93	91	3869
AbC283	124669	77	86	3648
AbC415	105563	102	95	2086
AbC554	97290	73	63	1006
AbC558	94506	127	142	906
AbC518	89488	94	69	800
AbC525	88864	108	99	682
AbC455	81285	125	125	2207
AbC291	77306	93	59	811
AbC453	72694	72	70	1682
AbC313	70815	57	75	583
AbC463	56824	65	58	599
AbC277	55027	59	78	534
AbC489	52204	82	98	450
AbC308	52166	80	74	637
AbC511	47575	86	93	473
AbC585	46098	81	75	519
AbC369	35698	56	35	187
AbC429	33945	56	78	256
AbC353	32474	33	15	275
AbC557	31256	70	62	220
AbC486	31103	88	118	317
AbC258	14343	81	83	155
AbC500	12901	43	64	107
AbC275	9170	42	17	131
AbC359	9166	30	19	57
AbC357	7101	47	35	94
AbC478	6805	46	21	91
AbC491	6235	32	23	69
AbC393	4423	301	886	167
AbC530	4315	47	45	28
AbC400	4191	250	5119	93
AbC561	2940	67	33	113

AbC310	1086	18	11	27
AbC574	1027	63	54	65
AbC298	866	331	61	104
AbC469	636	67	77	67
AbC285	569	5805	34	75

Notes: Antibodies were tested using direct assay format using U-PLEX assay. Briefly, AbC antibodies were labeled with SULFO-TAG, and added onto wells containing antigens directly linked onto each spot of U-PLEX plate. Antibodies are sorted based on ECL signal from trimeric S protein as antigen.

Table S4. Data used to generate Figure 1, demonstrating performance of 3 commercially available trimeric S glycoproteins screened via AbC525-AbC397 antibody pair.

			Trimeric S (Acro Biosystems)						Trimeric S (Sino Biological)						Trimeric S (BEI Resources)					
Capture Ab	Detection Ab	Conc (pg/mL)	Signal mean	SD	CV	% Recovery Mean	Calc. Conc. Mean	Calc. Conc. CV	Mean	Std. Deviation	CV	% Recovery Mean	Calc. Conc. Mean	Calc. Conc. CV	Mean	Std. Deviation	CV	% Recovery Mean	Calc. Conc. Mean	Calc. Conc. CV
AbC525	AbC397	1000	284934	1313.8	0.5	94.9	949.1	0.6	28610	3625.3	12.7	105.0	1049.8	13.4	1639	13.4	0.8	102.4	1024.4	0.9
AbC525	AbC397	250	122929	26271	21.4	127.8	319.6	26.3	7327	293.4	4.0	99.3	248.2	4.3	481	12.7	2.6	94.4	236.0	3.5
AbC525	AbC397	62.5	29605	1126.4	3.8	93.0	58.1	4.4	1949	33.9	1.7	94.4	59.0	1.9	211	39.6	18.8	108.9	68.1	34.5
AbC525	AbC397	15.625	8748	434.87	5.0	90.3	14.1	5.8	611	55.2	9.0	96.0	15.0	11.6	116	2.1	1.8	85.9	13.4	8.5
AbC525	AbC397	3.90625	2869	79.903	2.8	98.3	3.8	3.3	260	53.7	20.7	106.4	4.2	38.5	107	4.9	4.6	222.2	8.7	29.6
AbC525	AbC397	0.97656	1060	140.71	13.3	118.7	1.2	16.4	188	24.7	13.2	207.2	2.0	35.2	87	3.5	4.1	0.0	0.0	0.0
AbC525	AbC397	0.24414	321	7.0711	2.2	95.1	0.2	3.4	111	7.8	7.0	0.0	0.0	0.0	89	8.5	9.5	0.0	0.0	0.0
AbC525	AbC397	0	86			14.8			78			5.4			84			21.9		

Table S5. ECL signal and limit of detection (LOD) for the top ten performing AbC antibody pairs using trimeric S protein from Acro Biosystems. Antibody pairs are sorted by decreasing LOD.

Previous rank	Capture Ab	Detection Ab	Max signal (at 500 ng/mL)	Limit of detection (ng/mL)	Limit of detection (pg/mL)	Current rank
2	447	513	240769	0.058	58	2
6	447	518	255542	0.059	59	1
1	447	459	234199	0.074	74	3
4	525	447	198540	0.077	77	4
12	525	518	191901	0.079	79	5
7	525	513	164073	0.102	102	6
5	525	459	140986	0.127	127	7
34	447	447	127379	0.171	171	9
13	525	511	130791	0.204	204	8
42	447	511	88628	0.327	327	10

Table S6. The ECL signals from the top antibody candidates in round 1 paired with 3 antibodies from Sino Biologicals.

Capture Ab	Detection Ab	ECL signals at varying concentrations of trimer S protein									
		500 ng/mL	125 ng/mL	50 ng/mL	10 ng/mL	2 ng/mL	0.4 ng/mL	0.08 ng/mL	0.016 ng/mL	0.0032 ng/mL	0.0000
AbC447	MM43	474351	131285	26706	6043	1295	343	170	114	137	108
AbC447	D003	345577	92295	19026	4100	973	337	185	156	126	135
MM43	AbC447	247562	62576	13182	3026	826	346	334	353	282	257
MM43	D003	242611	65499	13153	2932	721	301	229	196	148	152
AbC447	AbC513	206360	57018	12200	2923	736	325	216	240	197	193
MM43	AbC513	191060	52926	10924	2780	1203	485	412	727	337	340
D003	MM43	190848	50287	10319	2523	639	270	185	221	181	183
MM43	AbC513	183836	50328	10787	2554	714	208	180	213	137	98
AbC447	AbC447	136347	34852	7678	1840	492	260	177	179	170	173
D003	AbC447	131881	33228	7234	1790	589	331	307	299	251	296
MM43	MM43	103416	26175	5082		352	156	166	143	184	90
D003	AbC513	58912	17384	3599	1077	552	357	296	565	406	336
D003	D003	49857	12273	2687	755	249	176	176	193	138	152
MM57	D003	47783	11273	2284	625	221	141	126	129	113	126
MM57	MM43	38732	9830	2112	560	185	152	112	144	113	117
MM57	AbC513	37500	10705	2420	647	246	150	140	179	162	148
MM57	AbC513	36224	9309	1996	543	236	127	121	130	134	118
MM57	AbC447	28101	6941	1554	439	221	147	161	168	143	143

Table S7. The ECL signals of AbCellera antibody pairs using 1000, 100, and 10 ng/mL of trimeric S protein antigen from Acros Biosystems. Antibody pairs are sorted at decreasing ECL signal at 100 ng/mL.

Antibody pairs		ECL signals at S protein concentrations		
Capture	Detection	1000 ng/mL	100 ng/mL	10 ng/mL
447	459	292818	45369	4549
447	513	288150	42124	4912
525	397	256264	31663	3222
525	447	276080	30948	3274
525	459	247874	30478	2965
447	518	223163	30334	3225
525	513	254434	29660	3352
447	283	211867	29082	3187
447	558	220547	28698	3047
447	415	191788	28491	3079
447	525	213091	27147	3096
525	518	212538	26360	2546
525	511	267397	25771	2839
353	447	255709	25413	2918
447	397	157582	25338	2733
277	447	239693	25241	2894
447	455	166083	23675	2775
447	277	172694	23644	2532
447	489	167814	22963	2677
447	554	165109	22099	2502
525	455	193566	21904	2409
353	397	195028	21761	2411
525	558	202904	21688	2309
525	308	192314	21573	2341
447	291	166560	21310	2444
518	447	194147	21033	2354
447	585	147245	20900	2198
525	489	192003	20725	2283
447	313	138181	19528	2142
525	277	160166	19146	1929
353	525	188417	18894	1997
313	447	190432	18575	2133
518	397	157746	18060	1975
447	447	153923	18039	2020
291	447	150398	17992	1999
277	397	163277	17899	1907
277	525	160169	17482	1871
313	397	158113	17293	1971
447	463	141262	17237	1945
525	453	148986	16964	1754
525	415	133459	16772	1688

447	511	115912	16289	2025
525	291	147959	15717	1697
447	453	105329	15450	1750
353	455	151760	15364	1904
525	554	139291	15339	1710
353	283	141425	14760	1695
518	525	142238	14253	1595
518	455	124324	14194	1617
291	397	114957	14139	1530
525	313	126535	14131	1576
277	455	124983	13442	1745
313	525	132607	13288	1476
353	429	139068	12855	1472
277	283	82140	12281	1379
291	525	115734	12192	1344
447	308	82818	12000	1401
353	585	117006	11979	1301
525	353	96336	11933	1167
525	463	135193	11384	1518
447	353	67867	11331	1034
518	283	105131	11187	1282
291	455	101959	11098	1430
585	447	111736	10996	1140
585	397	90026	10437	1167
277	585	93328	10121	1178
291	283	66183	9538	1120
353	511	92641	9534	1293
353	308	88931	9523	1075
313	283	86112	9505	1147
277	429	78460	9378	981
518	585	81226	9050	1018
277	511	84762	8951	1251
277	308	81278	8632	1010
291	429	86217	8183	1355
447	369	56400	8150	930
518	308	71736	7868	962
313	429	81005	7723	887
313	308	70623	7580	898
525	369	66721	7564	838
518	511	120679	7526	1013
558	447	65407	7253	814
585	459	62645	7186	778
291	585	65134	7133	866
561	447	67767	7021	935
558	397	53517	6675	747
518	429	69183	6601	732
291	308	62308	6408	750
585	455	57625	6225	741
291	511	58547	6209	937

478	459	52377	6166	682
561	397	55577	6150	696
525	429	74749	5879	583
585	518	53689	5794	638
585	513	54517	5789	708
313	585	54556	5737	725
313	455	56379	5679	840
369	447	59125	5522	819
561	525	57222	5504	730
486	447	49369	5448	717
478	513	47537	5359	726
585	308	51510	5281	643
561	283	45697	5277	653
585	558	50583	5163	603
313	511	50757	5140	769
585	511	93472	5125	699
415	447	44741	5008	713
558	525	47217	4887	616
585	489	47822	4727	607
478	518	31134	4551	429
463	447	41492	4470	580
558	283	39591	4470	560
585	291	44096	4401	549
486	397	39583	4387	556
369	397	35792	4379	478
558	455	37850	4366	548
486	525	42362	4288	561
369	525	39965	4172	599
561	429	35947	4049	501
561	455	37235	4042	586
585	453	34870	3986	444
463	397	35533	3985	505
558	429	33925	3938	460
585	313	36104	3927	468
486	455	33707	3865	561
478	447	34930	3817	494
585	277	34152	3791	450
369	455	34964	3657	643
415	397	33457	3654	468
561	585	33712	3594	515
459	447	31269	3547	472
478	558	37980	3522	504
486	283	30631	3511	462
585	554	31642	3279	386
478	397	31651	3257	411
283	447	31882	3189	474
585	463	35521	3175	404
585	353	27565	3142	356
585	415	27258	3053	357

478	525	34593	3021	438
558	308	25156	2995	415
561	308	26254	2954	440
463	525	29070	2931	417
447	429	24546	2922	376
283	513	25069	2837	456
310	513	26550	2831	435
463	455	25439	2820	418
558	585	25280	2779	398
310	447	25353	2756	424
486	585	23733	2754	398
291	453	27072	2751	407
369	283	31049	2711	493
561	511	58035	2691	509
429	459	19340	2675	330
369	511	24023	2609	513
310	455	24320	2580	427
369	585	25878	2579	404
415	525	25086	2578	440
478	415	23296	2527	327
459	397	21163	2511	339
478	283	26414	2497	340
558	511	28463	2482	402
478	277	21237	2477	351
463	283	21486	2376	355
310	459	21881	2370	327
283	397	19555	2366	291
369	429	20738	2365	365
415	455	20645	2358	414
459	455	19598	2352	370
283	455	20471	2305	373
429	558	18961	2275	362
459	283	18595	2257	314
369	308	22693	2218	357
478	554	21882	2204	345
310	511	18856	2188	404
486	429	19704	2171	316
429	447	18286	2142	365
478	489	21153	2115	360
486	511	64994	2109	371
478	585	22509	2097	294
283	511	17793	2090	350
459	525	19347	2057	344
585	369	16734	1978	241
415	283	17963	1965	316
486	308	16920	1956	318
478	463	21414	1924	285
310	415	16440	1894	256
463	585	16686	1885	267

585	429	18982	1875	289
308	447	17649	1865	337
310	558	15754	1828	325
283	558	15590	1819	303
308	525	15115	1817	280
310	518	18168	1803	355
429	513	15288	1791	289
283	459	15164	1782	261
463	308	14997	1744	293
310	489	18044	1708	344
283	518	14219	1655	279
429	525	13827	1647	270
478	291	19041	1632	282
478	313	16665	1567	253
415	308	14581	1562	241
463	511	73126	1543	313
415	429	13967	1512	277
310	283	13807	1493	262
283	489	13887	1491	276
310	554	14156	1482	265
415	511	12455	1481	335
429	415	11890	1473	205
283	308	14036	1467	249
310	525	12896	1460	255
310	463	14240	1446	238
429	489	11546	1446	287
277	453	16569	1439	250
310	308	13832	1423	261
518	518	2068	1387	170
310	313	13388	1376	197
463	429	12448	1359	247
459	511	53539	1357	240
429	291	12765	1346	214
310	585	12713	1344	233
308	459	12191	1334	252
478	369	12681	1332	249
459	585	10892	1322	259
459	308	10737	1320	214
429	313	11551	1313	212
415	585	10706	1311	216
310	291	12971	1305	212
478	353	11413	1305	178
308	283	15097	1299	289
310	277	12866	1299	216
429	585	9980	1287	213
429	283	13387	1275	238
429	397	9694	1271	220
283	554	11495	1241	243
429	554	11423	1225	231

283	291	11405	1213	258
308	489	10659	1202	303
429	518	10661	1199	271
310	453	10788	1194	222
283	415	8831	1171	227
283	313	10439	1158	218
459	453	9373	1154	215
308	513	9871	1141	213
308	558	9845	1125	290
310	429	9442	1116	220
283	463	9933	1076	226
308	397	8957	1069	211
308	585	9871	1054	185
429	463	8672	1001	204
486	453	8174	991	212
283	453	9187	987	224
313	453	9570	965	197
478	511	66747	943	266
283	277	8904	937	234
308	291	8170	889	162
308	554	9145	881	221
518	459	10428	880	188
459	429	7179	879	172
308	313	7845	878	136
308	518	7765	864	219
310	397	7124	854	166
308	415	7164	824	148
459	513	6346	790	181
429	277	8200	776	167
429	353	6539	771	151
310	353	6526	757	157
283	353	4602	752	175
459	459	6223	744	157
518	513	9156	743	204
277	478	8748	739	172
353	453	7919	698	170
558	453	5911	674	182
308	277	5989	662	181
308	463	5891	651	167
277	459	8253	648	172
283	369	5777	648	179
429	369	6344	576	181
283	429	6727	566	158
478	308	4916	556	160
525	283	7190	532	187
313	459	6657	523	161
310	369	4154	493	126
277	513	8484	477	172
415	459	3918	467	128

415	513	4130	467	161
308	453	4050	452	145
291	459	4679	446	132
525	525	5223	441	199
313	513	6026	429	184
518	415	4530	429	145
518	453	4787	428	147
459	415	3239	427	129
558	459	3089	425	142
486	459	3151	419	143
561	453	3092	419	140
463	518	873	412	151
486	513	2993	412	161
561	513	3141	400	152
558	513	3089	394	178
463	513	2413	391	170
459	558	2476	386	150
353	478	5315	385	176
353	459	4362	384	199
291	513	4635	376	155
308	353	3091	376	135
561	459	3125	373	168
308	369	3669	364	111
463	459	2529	361	143
478	455	1518	358	134
518	558	3978	343	194
585	283	2761	338	151
459	518	2016	327	131
459	489	1785	323	140
369	513	2010	322	156
415	415	2328	318	117
353	513	5112	316	129
277	415	3066	315	139
313	415	2881	314	135
415	453	2073	312	114
459	313	2191	300	100
283	283	6563	299	120
429	429	542	296	123
459	554	2287	295	152
291	415	2214	294	144
459	291	2306	291	118
558	415	2034	289	121
518	489	1785	286	197
561	558	1968	285	204
277	558	3529	283	144
518	291	3933	283	131
463	415	1557	277	121
369	459	2035	274	125
518	313	3672	271	140

369	558	1117	267	183
486	558	1677	267	138
283	525	1355	262	150
277	313	2864	261	113
585	525	1796	258	136
429	511	1345	256	144
277	291	3026	255	120
561	518	1142	255	190
369	518	543	253	184
518	554	2994	253	133
277	489	1403	252	185
561	489	1367	251	181
561	415	1646	250	112
558	558	1189	249	128
369	478	1492	247	134
459	463	1838	247	108
463	453	1457	247	147
463	558	1129	247	145
369	453	2062	245	86
478	453	1031	244	129
486	415	1797	242	110
486	489	975	241	149
558	518	853	240	108
291	478	2294	239	102
415	558	1556	238	147
459	277	1545	237	120
518	463	2621	237	120
353	415	1804	235	117
415	554	1391	232	92
561	554	1427	226	111
277	518	1349	223	142
463	489	821	219	154
353	489	810	216	251
415	489	880	215	165
561	291	1684	215	116
429	455	907	214	115
561	313	1474	214	108
561	463	1222	214	111
353	554	1418	212	115
353	558	2069	209	194
415	313	1370	208	87
277	554	2413	207	184
558	489	743	205	129
313	291	1929	204	144
459	353	896	202	99
415	291	1278	201	96
415	463	1119	201	116
291	489	698	200	123
353	313	1760	200	109

558	313	949	199	127
308	455	923	197	165
313	558	2057	197	155
353	291	2186	197	96
558	291	1069	197	116
518	353	1090	195	100
353	518	754	194	197
291	554	1276	193	139
525	585	1666	193	146
585	585	860	193	136
313	554	1738	191	126
463	554	1014	191	129
369	489	624	189	249
486	463	1006	189	111
313	478	1694	188	103
463	313	976	188	119
313	313	1864	187	131
369	415	849	187	89
518	277	1271	186	127
291	291	1464	183	118
310	478	880	182	94
478	429	528	182	128
277	463	2258	180	129
486	554	1186	179	141
486	313	1094	178	118
561	277	804	176	123
291	463	1154	174	92
429	308	757	174	120
486	518	1085	174	132
558	554	1092	173	124
291	518	729	172	124
291	558	1662	172	130
308	511	522	171	180
313	463	1531	170	116
415	518	1021	170	130
561	353	666	170	122
353	463	1230	166	118
369	313	855	166	88
463	277	602	164	133
486	291	1273	164	120
369	463	644	163	93
353	277	615	162	116
369	554	814	162	143
277	277	934	161	160
291	313	1445	161	121
313	277	705	161	135
283	478	579	159	107
283	585	670	159	129
463	463	834	159	122

558	463	861	159	118
313	489	905	157	165
558	277	532	156	100
415	277	651	155	101
313	518	928	154	161
415	478	645	154	123
463	291	1028	153	119
308	308	500	151	109
369	291	902	150	93
463	353	469	149	91
277	353	791	148	74
291	369	299	141	99
486	277	690	141	137
291	277	574	138	119
486	353	465	137	106
369	369	233	135	81
518	369	541	135	122
353	353	549	133	141
369	277	334	133	109
429	453	565	131	88
308	429	534	130	124
313	369	364	125	121
463	369	307	125	92
369	353	384	124	123
415	369	303	122	109
277	369	452	120	111
429	478	191	120	110
558	353	414	120	91
415	353	498	119	114
561	369	344	119	72
353	369	323	116	101
308	478	288	106	109
313	353	573	104	121
291	353	432	100	93
558	369	293	99	102
486	369	279	87	112
459	369	598	78	102

Table S8. The ECL from the round 3 top antibody pairs and detection of SARS-CoV-2 virions.

Capture Ab	Detection Ab	Ag	Dilution	Concentration (pg/mL)	GE/mL	Mean	Adj. Sig. Mean	CV	% Recovery Mean	Calc. Conc. Mean	Calc. Conc. CV
L2355	L2215	AB		625		2529633	2529525.0	1.6	96.6	603.8	3.0
L2355	L2215	AB		62.5		569410	569302.5	3.8	116.1	72.6	4.5
L2355	L2215	AB		6.25		60865	60757.0	0.7	94.6	5.9	0.7
L2355	L2215	AB		1.25		13167	13059.5	2.3	89.6	1.1	2.5
L2355	L2215	AB		0.25		3518	3410.5	1.8	104.6	0.3	2.0
L2355	L2215	AB		0.05		924	816.0	6.0	107.8	0.1	7.7
L2355	L2215	AB		0.01		316	208.5	0.0	104.4	0.0	0.0
L2355	L2215	AB		0.002		180	72.0	5.9	87.9	0.0	35.4
L2355/	L2215	CoV-2	1	100000	6.07E+08	246224	246116.5	5.2		27.6	5.9
L2355	L2215	CoV-2	5	20000	1.21E+08	38153	38045.5	2.7		17.8	2.9
L2355	L2215	CoV-2	25	4000		6948	6840.0	1.0		13.9	1.1
L2355	L2215	CoV-2	125	800	0.00E+00	1670	1562.5	9.7		13.9	11.5
L2355	L2215	CoV-2	625	160	0.00E+00	563	455.5	0.5		17.2	0.7
L2355	L2215	CoV-2	3125	32	0.00E+00	242	134.0	3.8		17.4	10.5
L2355	L2215	CoV-2	15625	6.4	0.00E+00	187	79.5	1.5		34.4	7.7
L2355	L2215	CoV-2	78125	1.28	0.00E+00	133	25.0	12.3		0.0	0.0
L2355	L2215	CoV	0.1	10000		150	42.5	4.7		0.0	0.0
L2355	L2215	CoV	0.5	2000		126	18.5	6.7		0.0	0.0
L2355	L2215	CoV	2.5	400		158	50.5	3.6		0.0	57.1
L2355	L2215	MERS	0.1	10000		108	0.5	10.5		0.0	0.0
L2355	L2215	MERS	0.5	2000		146	38.5	6.8		0.0	0.0
L2355	L2215	hCoV	0.1	10000		123	15.0	11.0		0.0	0.0
L2355	L2215	hCoV	0.5	2000		147	39.0	8.2		0.0	0.0

L2381	L2215	AB		625		2027036	2026903.5	1.1	98.2	613.55	1.8
L2381	L2215	AB		62.5		369106	368974.0	2.0	110.6	69.10	2.2
L2381	L2215	AB		6.25		39220	39087.5	2.9	96.2	6.01	3.1
L2381	L2215	AB		1.25		8229	8097.0	0.0	89.9	1.12	0.0
L2381	L2215	AB		0.25		2248	2116.0	1.1	106.7	0.27	1.3
L2381	L2215	AB		0.05		641	509.0	9.7	110.5	0.06	14.0
L2381	L2215	AB		0.01		260	128.0	9.8	94.8	0.01	30.3
L2381	L2215	AB		0.002		190	58.0	10.4	93.8	0.00	107.8
L2381/	L2215	CoV-2	1	100000	6.07E+08	56300	56167.5	5.6		8.86	6.0
L2381	L2215	CoV-2	5	20000	1.21E+08	10820	10687.5	1.1		7.55	1.2
L2381	L2215	CoV-2	25	4000	2.43E+07	2441	2309.0	5.4		7.33	6.1
L2381	L2215	CoV-2	125	800	4.86E+06	661	529.0	5.6		7.21	8.0
L2381	L2215	CoV-2	625	160	9.71E+05	295	163.0	17.3		8.43	43.4
L2381	L2215	CoV-2	3125	32	1.94E+05	184	52.0	13.8		0.00	0.0
L2381	L2215	CoV-2	15625	6.4	3.88E+04	152	20.0	6.5		0.00	0.0
L2381	L2215	CoV-2	78125	1.28	7.77E+03	141	8.5	27.7		0.00	0.0
L2381	L2215	CoV	0.1	10000		99	-33.0	22.9		0.0	0.0
L2381	L2215	CoV	0.5	2000		127	-5.5	6.1		0.0	0.0
L2381	L2215	CoV	2.5	400		129	-3.0	6.6		0.0	0.0
L2381	L2215	MERS	0.1	10000		148	15.5	5.3		0.0	0.0

L2381	L2215	MERS	0.5	2000		122	-10.5	4.1		0.0	0.0
L2381	L2215	hCoV	0.1	10000		119	-13.5	10.1		0.0	0.0
L2381	L2215	hCoV	0.5	2000		158	25.5	6.7		0.0	0.0

L2838	L2215	AB		625		2634204	2634054.0	0.3	95.8	598.46	0.7
L2838	L2215	AB		62.5		648553	648403.0	3.4	115.5	72.21	4.0
L2838	L2215	AB		6.25		67405	67255.5	5.0	95.1	5.94	5.3
L2838	L2215	AB		1.25		13887	13737.5	1.1	89.4	1.12	1.2
L2838	L2215	AB		0.25		3648	3498.0	2.3	105.4	0.26	2.6
L2838	L2215	AB		0.05		1003	853.5	7.5	112.9	0.06	10.0
L2838	L2215	AB		0.01		363	213.5	9.4	95.5	0.01	24.8
L2838	L2215	AB		0.002		250	100.5	18.1	0.0	0.00	0.0
L2838	L2215	CoV-2	1	100000	6.07E+08	182667	182517.0	6.4		17.3	6.9
L2838	L2215	CoV-2	5	20000	1.21E+08	32977	32827.0	0.5		14.0	0.5
L2838	L2215	CoV-2	25	4000	2.43E+07	6634	6484.5	2.1		12.7	2.3
L2838	L2215	CoV-2	125	800	4.86E+06	1695	1545.0	6.9		13.7	8.3
L2838	L2215	CoV-2	625	160	9.71E+05	583	433.0	1.1		15.8	1.8
L2838	L2215	CoV-2	3125	32	1.94E+05	268	118.0	1.9		9.4	11.0
L2838	L2215	CoV-2	15625	6.4	3.88E+04	217	67.5	10.4		0.0	0.0
L2838	L2215	CoV-2	78125	1.28	7.77E+03	157	7.5	5.4		0.0	0.0
L2838	L2215	CoV	0.1	10000		188	38.5	9.8		0.0	0.0
L2838	L2215	CoV	0.5	2000		145	-4.5	17.6		0.0	0.0
L2838	L2215	CoV	2.5	400		194	44.5	1.5		0.0	0.0
L2838	L2215	MERS	0.1	10000		143	-7.0	4.5		0.0	0.0
L2838	L2215	MERS	0.5	2000		167	17.0	16.6		0.0	0.0
L2838	L2215	hCoV	0.1	10000		167	17.0	8.1		0.0	0.0
L2838	L2215	hCoV	0.5	2000		178	28.5	4.8		0.0	0.0

Table S9. Data for Table 3.

Number used for MSD testing	Biorepository ID	Type	COVID status as reported to PATH	N gene Ct value reported to PATH	ORF1 gene Ct value reported to PATH	S gene Ct value reported to PATH	S conc, ng/mL	S conc, ng/mL (Adj)	Detection Range	Diagnostic performance
14	FIL-0029-NU-01	NU	Negative	NA	NA	NA	0.00	0.00	Below Fit Curve Range	TN
16	FIL-0032-NU-01	NU	Negative	NA	NA	NA	0.00	0.00	Below Fit Curve Range	TN
17	FIL-0033-NU-01	NU	Negative	NA	NA	NA	0.00	0.00	Below Fit Curve Range	TN
18	FIL-0115-NV-02	NB	Negative	NA	NA	NA	0.00	0.00	Below Fit Curve Range	TN
22	FIL-1010-NP-4	NP	Negative	NA	NA	NA	0.00	0.00	Below Fit Curve Range	TN
23	FIL-1012-NP-4	NP	Negative	NA	NA	NA	0.00	0.00	Below Fit Curve Range	TN
24	FIL-1019-NP-3	NP	Negative	NA	NA	NA	0.00	0.00	Below Fit Curve Range	TN
25	FIL-1020-NP-3	NP	Negative	NA	NA	NA	0.00	0.00	Below Fit Curve Range	TN
26	FIL-1021-NP-3	NP	Negative	NA	NA	NA	0.00	0.00	Below Fit Curve Range	TN
39	FIL-0022-NU-02	NU	Negative	NA	NA	NA	0.00	0.00	Below Fit Curve Range	TN
40	FIL-0023-NU-02	NU	Negative	NA	NA	NA	0.00	0.00	Below Fit Curve Range	TN
41	FIL-0024-NU-02	NU	Negative	NA		NA	0.00	0.00	Below Fit Curve Range	TN
42	FIL-0025-NU-02	NU	Negative	NA	NA	NA	0.00	0.00	Below Fit Curve Range	TN
43	FIL-0026-NU-01	NU	Negative	NA	NA	NA	0.00	0.00	Below Fit Curve Range	TN
44	FIL-0131-NB-02	NB	Negative	NA	NA	NA	0.00	0.00	Below Fit Curve Range	TN
49	FIL-1013-NP-3	NP	Negative	NA	NA	NA	0.00	0.00	Below Fit Curve Range	TN
50	FIL-1014-NP-3	NP	Negative	NA	NA	NA	0.00	0.00	Below Fit Curve Range	TN
51	FIL-1016-NP-3	NP	Negative	NA	NA	NA	0.00	0.00	Below Fit Curve Range	TN
52	FIL-1017-NP-2	NP	Negative	NA	NA	NA	0.00	0.00	Below Fit Curve Range	TN
53	FIL-1018-NP-3	NP	Negative	NA	NA	NA	0.00	0.00	Below Fit Curve Range	TN
1	NWP-0001-NP-01	NP	Positive	NR	NR	NR	0.01	0.01	Below Detection Range	FN
2	NWP-0002-NP-01	NP	Positive	NR	NR	NR	0.11	0.11	In Detection Range	TP
3	NWP-0003-NP-01	NP	Positive	NR	NR	NR	0.39	0.39	In Detection Range	TP
4	NWP-0001-NP-02	NP	Positive	NR	NR	NR	0.00	0.00	Below Fit Curve Range	FN
5	NWP-0002-NP-02	NP	Positive	NR	NR	NR	0.02	0.02	In Detection Range	TP
6	NWP-0003-NP-02	NP	Positive	NR	NR	NR	0.03	0.03	In Detection Range	TP
7	NWP-0001-NP-03	NP	Positive	NR	NR	NR	0.00	0.00	Below Detection Range	FN
8	NWP-0002-NP-03	NP	Positive	NR	NR	NR	0.07	0.07	In Detection Range	TP
9	NWP-0003-NP-03	NP	Positive	NR	NR	NR	3.49	3.49	In Detection Range	TP
10	NWP-0001-NP-04	NP	Positive	NR	NR	NR	0.19	0.19	In Detection Range	TP
11	NWP-0002-NP-04	NP	Positive	NR	NR	NR	0.00	0.00	Below Fit Curve Range	FN
12	NWP-0003-NP-04	NP	Positive	NR	NR	NR	0.01	0.01	Below Detection Range	FN
13	NWP-0001-NP-05	NP	Positive	NR	NR	NR	0.00	0.00	Below Fit Curve Range	FN
15	FIL-0030-NU-02	NU	Positive	18.3	18.1	19.4	0.82	0.82	In Detection Range	TP
19	FIL-0180-NU-03	NU	Positive	32.5	30.9	30.3	0.00	0.00	Below Detection Range	FN
20	FIL-0662-NU-3	NU	Positive	26.5	24.8	25.7	0.07	0.07	In Detection Range	TP
21	FIL-0684-NU-3	NU	Positive	19.8	18.8	18.9	9.81	9.81	In Detection Range	TP
27	FIL-1212-NU-3	NU	Positive	28.9	27.5	27.8	0.04	0.04	In Detection Range	TP
28	FIL-1326-NU-02	NU	Positive	25.8	25.1	25.7	0.00	0.00	Below Detection Range	FN
29	FIL-2155-NP-03	NP	Positive	25.1	24.5	24.8	0.20	0.20	In Detection Range	TP
30	FIL-2156-NP-03	NP	Positive	21.5	20.9	21.3	1.49	1.49	In Detection Range	TP
31	FIL-2162-NP-03	NP	Positive	17.8	17.5	17.6	21.77	21.77	In Detection Range	TP
32	FIL-2164-NP-03	NP	Positive	19.7	19.0	19.2	3.76	3.76	In Detection Range	TP
33	FIL-2165-NP-03	NP	Positive	22.0	21.6	21.8	2.71	2.71	In Detection Range	TP
34	FIL-2167-NP-02	NP	Positive	18.1	17.5	17.7	20.34	20.34	In Detection Range	TP
35	FIL-2186-NP-02	NP	Positive	18.3	18.8	19.1	5.61	5.61	In Detection Range	TP
36	FIL-2191-NP-02	NP	Positive	15.8	16.2	16.1	55.21	55.21	In Detection Range	TP
37	FIL-2196-NP-02	NP	Positive	19.5	19.3	19.4	6.71	6.71	In Detection Range	TP
38	FIL-2197-NP-02	NP	Positive	21.9	20.8	20.8	1.55	1.55	In Detection Range	TP
45	FIL-0133-NB-02	NB	Positive	16.8	16.2	16.2	13.81	13.81	In Detection Range	TP
46	FIL-0134-NB-02	NB	Positive	21.3	21.0	21.2	0.65	0.65	In Detection Range	TP
47	FIL-0167-NU-03	NU	Positive	19.6	17.9	18.0	3.84	3.84	In Detection Range	TP
48	FIL-0179-NU-03	NU	Positive	23.7	23.5	24.0	0.00	0.00	Below Fit Curve Range	FN

LOD		0.01		

TP	TP = true positive	24
TN	TN = true negative	20
FP	FP = false positive	0
FN	FN = false negative	9
Total		53
Sens	Sen = TP/(TP+FN)	0.73
Spec	Spe = TN/(TN+FP)	1.00
PPV	PPV = TP/(TP+FP)	1.00
NPV	NPV = TN/(TN+FN)	0.69

Table S10. Biotinylation efficiencies for each antibody used as a capture reagent in liquid immunoassay.

Antibodies	Lot no.	IgG conc (mg/mL)	HABA/ Avidin absorbance before sample addition	HABA/ Avidin absorbance after sample addition	Change in absorbance at 500 nm	IgG (mmol/mL)*	Conc biotin (mmol/ml)	mmol biotin/mmol mAb*
AC275 bio	JC200505	0.873	0.594	0.574	0.020	0.000006	0.000001	8.1
AC277 bio	JC200505	0.984	0.592	0.561	0.031	0.000007	0.000002	11.0
AC283 bio	JC200505	1.028	0.602	0.565	0.037	0.000007	0.000002	12.9
AC285 bio	JC200505	0.988	0.608	0.567	0.041	0.000007	0.000002	14.7
AC291 bio	JC200505	0.971	0.599	0.561	0.038	0.000006	0.000002	13.8
AC308 bio	JC200505	0.925	0.593	0.568	0.025	0.000006	0.000001	9.7
AC310 bio	JC200505	1.061	0.598	0.568	0.030	0.000007	0.000002	10.1
AC313 bio	JC200505	0.944	0.605	0.573	0.033	0.000006	0.000002	12.2
AC353 bio	JC200505	1.140	0.605	0.583	0.022	0.000008	0.000001	6.8
AC357 bio	JC200505	0.755	0.594	0.560	0.034	0.000005	0.000002	16.0
AC359 bio	JC200505	1.022	0.591	0.556	0.035	0.000007	0.000002	12.2
AC369 bio	JC200505	0.943	0.600	0.564		0.000006	0.000000	0.0
AC397 bio	JC200505	0.903	0.593	0.579	0.014	0.000006	0.000001	5.3
AC415 bio	JC200505	0.947	0.601	0.564	0.038	0.000006	0.000002	14.0
AC429 bio	JC200505	0.967	0.592	0.561	0.031	0.000006	0.000002	11.4
AC453 bio	JC200505	0.829	0.617	0.576	0.041	0.000006	0.000002	17.6
AC459 bio	JC200505	0.927	0.588	0.561	0.026	0.000006	0.000002	10.1
AC463 bio	JC200505	0.952	0.566	0.541	0.025	0.000006	0.000001	9.2
AC478 bio	JC200505	0.908	0.595	0.566	0.030	0.000006	0.000002	11.6
AC489 bio	JC200505	0.942	0.615	0.584	0.031	0.000006	0.000002	11.6
AC491 bio	JC200505	0.869	0.595	0.562	0.033	0.000006	0.000002	13.6
AC500 bio	JC200505	0.980	0.618	0.583	0.034	0.000007	0.000002	12.3
AC530 bio	JC200505	1.020	0.598	0.569	0.029	0.000007	0.000002	10.1
AC554 bio	JC200505	0.729	0.615	0.572	0.043	0.000005	0.000003	20.9
AC258 bio	JC200513	0.871	0.582	0.556	0.026	0.000006	0.000002	10.6
AC298 bio	JC200513	1.080	0.583	0.614	-0.031	0.000007	-0.000002	-10.2
AC298 bio	JC200527	0.264	0.549	0.539	0.009	0.000002	0.000001	12.6
AC393 bio	JC200513	1.035	0.597	0.567	0.031	0.000007	0.000002	10.5
AC400 bio	JC200513	1.023	0.590	0.559	0.031	0.000007	0.000002	10.5
AC447 bio	JC200513	0.956	0.592	0.549	0.043	0.000006	0.000003	16.0
AC447 bio	JC200710E	0.912	0.559	0.542	0.017	0.000006	0.000001	6.5
AC455 bio	JC200513	0.747	0.599	0.572	0.027	0.000005	0.000002	12.9
AC469 bio	JC200513	1.103	0.593	0.576	0.017	0.000007	0.000001	5.4
AC486 bio	JC200513	0.969	0.588	0.557	0.031	0.000006	0.000002	11.2
AC511 bio	JC200513	1.090	0.568	0.543	0.025	0.000006	0.000001	9.1
AC513 bio	JC200513	1.079	0.566	0.538	0.028	0.000007	0.000002	9.2
AC518 bio	JC200513	1.048	0.580	0.556	0.023	0.000007	0.000001	7.8

AC525 bio	JC200513	0.977	0.583	0.572	0.011	0.000007	0.000001	3.9
AC557 bio	JC200513	0.955	0.588	0.566	0.022	0.000006	0.000001	8.2
AC558 bio	JC200513	0.993	0.591	0.568	0.023	0.000007	0.000001	8.1
AC561 bio	JC200513	0.937	0.593	0.573	0.020	0.000006	0.000001	7.4
AC574 bio	JC200513	0.942	0.592	0.568	0.024	0.000006	0.000001	9.0
AC585 bio	JC200513	1.005	0.601	0.563	0.038	0.000007	0.000002	13.2
L2215-bio	JC200710A	0.896	0.554	0.537	0.016	0.000006	0.000001	6.4
L2355-bio	JC200710B	0.898	0.553	0.534	0.019	0.000006	0.000001	7.5
L2381-bio	JC200710C	0.805	0.559	0.549	0.010	0.000005	0.000001	4.2
L2838-bio	JC200710D	0.992	0.553	0.537	0.016	0.000007	0.000001	5.7
SBMM43-bio	JC200609	0.463	0.600	0.565	0.034	0.000003	0.000002	26.0
SBMM57-bio	JC200527	0.428	0.572	0.550	0.022	0.000003	0.000001	18.0
SBD003-bio	JC200527	0.248	0.571	0.561	0.011	0.000002	0.000001	15.2

*calculated assuming that MW of each antibody = 150,000 Da

Min	-10.2
Max	26.0
Ave	10.4

Table S11. The conjugation efficiency of the SULFO-TAG for each antibody used as the detector reagent in the liquid immunoassay.

Sample ID	Lot #	IgG conc (mg/mL)	IgG (M) *	Sulfo-tag conc avg (µM)	Sulfo-tag label:IgG
AC275_ST	JC200504	1.048	6.98E-06	48.5	6.9
AC277_ST	JC200504	1.073	7.15E-06	50.3	7.0
AC283_ST	JC200504	1.025	6.83E-06	51.7	7.6
AC285_ST	JC200504	1.111	7.41E-06	52.5	7.1
AC291_ST	JC200504	1.215	8.10E-06	47.6	5.9
AC308_ST	JC200504	1.054	7.02E-06	45.8	6.5
AC310_ST	JC200504	1.114	7.43E-06	56.0	7.5
AC313_ST	JC200504	1.105	7.37E-06	38.1	5.2
AC353_ST	JC200504	1.005	6.70E-06	32.0	4.8
AC357_ST	JC200504	0.904	6.03E-06	49.5	8.2
AC359_ST	JC200504	1.017	6.78E-06	51.6	7.6
AC369_ST	JC200504	0.934	6.23E-06	30.1	
AC397_ST	JC200504	0.936	6.24E-06	44.4	7.1
AC415_ST	JC200504	1.009	6.73E-06	45.0	6.7
AC429_ST	JC200504	1.052	7.01E-06	71.7	10.2
AC453_ST	JC200504	1.081	7.21E-06	46.1	6.4
AC459_ST	JC200504	0.987	6.58E-06	47.5	7.2
AC463_ST	JC200504	0.875	5.83E-06	33.7	5.8
AC478_ST	JC200504	0.945	6.30E-06	43.5	6.9
AC489_ST	JC200504	0.848	5.65E-06	58.5	10.3
AC491_ST	JC200504	0.993	6.62E-06	37.0	5.6
AC500_ST	JC200504	1.005	6.70E-06	44.9	6.7
AC530_ST	JC200504	0.978	6.52E-06	48.4	7.4
AC554_ST	JC200504	0.895	5.97E-06	34.6	5.8
AC258_ST	JC200513	0.938	6.25E-06	55.9	8.9
AC298_ST	JC200513	0.958	6.38E-06	62.6	9.8
AC393_ST	JC200513	0.975	6.50E-06	61.3	9.4
AC400_ST	JC200513	0.920	6.14E-06	56.0	9.1
AC447_ST	JC200513	0.897	5.98E-06	44.6	7.5
AC455_ST	JC200513	0.686	4.57E-06	50.5	11.0
AC469_ST	JC200513	0.975	6.50E-06	66.3	10.2
AC486_ST	JC200513	1.048	6.98E-06	57.4	8.2
AC511_ST	JC200513	1.008	6.72E-06	59.3	8.8
AC513_ST	JC200513	0.949	6.33E-06	52.8	8.3
AC518_ST	JC200513	1.008	6.72E-06	61.4	9.1
AC525_ST	JC200513	0.821	5.48E-06	56.1	10.2
AC557_ST	JC200513	1.092	7.28E-06	58.9	8.1
AC558_ST	JC200513	1.014	6.76E-06	57.6	8.5
AC561_ST	JC200513	0.963	6.42E-06	56.0	8.7
AC574_ST	JC200513	0.982	6.55E-06	61.4	9.4
AC585_ST	JC200513	0.883	5.88E-06	53.4	9.1
L2251-ST	JC200710C	0.891	5.94E-06	56.1	9.4
L2355-ST	JC200710D	1.109	7.39E-06	68.6	9.3

L2381-ST	JC200710E	1.005	6.70E-06	66.9	10.0
L2838-ST	JC200710F	1.097	7.31E-06	68.1	9.3
SBD003_ST	JC200527	0.426	2.84E-06	18.3	6.4
SBMM43_ST	JC200609	0.555	3.70E-06	30.7	8.3
SBMM57_ST	JC200527	0.943	6.29E-06	52.8	8.4

*calculated assuming that MW of each antibody = 150,000 Da

Min	4.8
Max	11.0
Ave	8.0
